# Cellular and Pectin Dynamics during Abscission Zone Development and Ripe Fruit Abscission of the Monocot Oil Palm

**DOI:** 10.3389/fpls.2016.00540

**Published:** 2016-04-26

**Authors:** Peerapat Roongsattham, Fabienne Morcillo, Kim Fooyontphanich, Chatchawan Jantasuriyarat, Somvong Tragoonrung, Philippe Amblard, Myriam Collin, Gregory Mouille, Jean-Luc Verdeil, Timothy J. Tranbarger

**Affiliations:** ^1^UMR DIADE, Institut de Recherche pour le DéveloppementMontpellier, France; ^2^UMR DIADE, CIRADMontpellier, France; ^3^Department of Genetics, Kasetsart UniversityBangkok, Thailand; ^4^National Center for Genetic Engineering and Biotechnology, Genome InstitutePathum Thani, Thailand; ^5^PalmElit SASMontferrier-sur-Lez, France; ^6^Institut Jean-Pierre Bourgin, UMR1318 Institut National de la Recherche Agronomique -AgroParisTechERL3559 Centre National de la Recherche Scientifique, France; ^7^UMR AGAP, PHIV-MRI CIRADMontpellier, France

**Keywords:** fruit abscission, abscission zone, pectin, oil palm, cell wall

## Abstract

The oil palm (*Elaeis guineensis* Jacq.) fruit primary abscission zone (AZ) is a multi-cell layered boundary region between the pedicel (P) and mesocarp (M) tissues. To examine the cellular processes that occur during the development and function of the AZ cell layers, we employed multiple histological and immunohistochemical methods combined with confocal, electron and Fourier-transform infrared (FT-IR) microspectroscopy approaches. During early fruit development and differentiation of the AZ, the orientation of cell divisions in the AZ was periclinal compared with anticlinal divisions in the P and M. AZ cell wall width increased earlier during development suggesting cell wall assembly occurred more rapidly in the AZ than the adjacent P and M tissues. The developing fruit AZ contain numerous intra-AZ cell layer plasmodesmata (PD), but very few inter-AZ cell layer PD. In the AZ of ripening fruit, PD were less frequent, wider, and mainly intra-AZ cell layer localized. Furthermore, DAPI staining revealed nuclei are located adjacent to PD and are remarkably aligned within AZ layer cells, and remain aligned and intact after cell separation. The polarized accumulation of ribosomes, rough endoplasmic reticulum, mitochondria, and vesicles suggested active secretion at the tip of AZ cells occurred during development which may contribute to the striated cell wall patterns in the AZ cell layers. AZ cells accumulated intracellular pectin during development, which appear to be released and/or degraded during cell separation. The signal for the JIM5 epitope, that recognizes low methylesterified and un-methylesterified homogalacturonan (HG), increased in the AZ layer cell walls prior to separation and dramatically increased on the separated AZ cell surfaces. Finally, FT-IR microspectroscopy analysis indicated a decrease in methylesterified HG occurred in AZ cell walls during separation, which may partially explain an increase in the JIM5 epitope signal. The results obtained through a multi-imaging approach allow an integrated view of the dynamic developmental processes that occur in a multi-layered boundary AZ and provide evidence for distinct regulatory mechanisms that underlie oil palm fruit AZ development and function.

## Introduction

Plant organ abscission is a highly regulated developmental process that results in the loss of various organs throughout the life cycle of the plant. The abscission process is complex with many overlapping points of regulation, and involves the integration of multiple external and internal signals that depend on the overall status of the plant (Roberts et al., 2002; Estornell et al., 2013). In particular, seed and fruit abscission are important to coordinate seed dispersal for plant reproductive success. If fruit are shed prematurely before seed development is complete, or too late in relation to seasonal climate changes, reproductive success can be jeopardized. For crop species, seed and fruit abscission are important traits to consider, if seed or fruit abscission occur too early or late, the economic consequences can be significant.

Central to the process is the role of the abscission zone (AZ) where cell separation occurs that leads to organ detachment. Cell separation that occurs during abscission is a tissue and cellular process, which involves the differentiation of the AZ located at the base of the organ to be shed. Generally, the plant AZ consists of one to several cell layers but can also consist of multiple layers as observed in the leaflet rachis of *Sambucus nigra* (common elder) with up to 30–40 layers (Osborne and Sargent, 1976). AZ cells are typically isodiametrically shaped with dense cytoplasms (Addicott, 1982; Sexton and Roberts, 1982; Roberts et al., 2002). Neighboring cells are joined together by the middle lamella composed primarily of pectin, the most structurally complex family of cell wall polysaccharides and a major component of primary walls of both monocots and dicots (Mohnen, 2008). Pectin, principally homogalacturonan (HG), is also the main component of the middle lamella between adjacent cells and is of paramount importance for cell adhesion and during cell separation (Willats et al., 2001a; Jarvis et al., 2003; Ogawa et al., 2009; Iwai et al., 2013; Daher and Braybrook, 2015). In addition, pectin derived oligogalacturonide degradation products can also act as signaling molecules, possibly through the action of ethylene (Baldwin and Biggs, 1988; Brecht and Huber, 1988; Campbell and Labavitch, 1991; Melotto et al., 1994; Ridley et al., 2001). However, the structural characteristics of pectin and how it functions during cell separation underlying organ abscission are not completely understood.

The methylesterification of HG plays an important role during plant development, can modulate the functionality of pectin, in particular for cell adhesion and for cell separation to occur (Willats et al., 2001b; Jarvis et al., 2003; Mouille et al., 2007). HG is thought to be synthesized in the Golgi complex, targeted through vesicles *via* the plasma membrane to the apoplast and finally inserted into the cell wall in a highly methylesterified form (Zhang and Staehelin, 1992; Atmodjo et al., 2013). After cell divisions, pectin undergoes demethylesterification at cell junctions where cell separation takes place for intercellular space formation (Willats et al., 2001b; Jarvis et al., 2003). The demethylesterification of HG is catalyzed by pectin methylesterases (PME, EC 3.1.1.11), which modulate HG methylation status and consequently plant development (Wolf et al., 2009). It is believed that demethylesterification allows the formation of calcium (Ca^2+^) cross-links and the “egg-box” pectin configuration between adjacent HG polymers, which can lead to the formation of rigid pectin gels or HG degradation by pectin degrading polygalacturonases (PGs, EC 3.2.1.15) (Grant et al., 1973; Cosgrove, 2005; Senechal et al., 2014). Indeed, PGs modify the texture and rigidity of the cell wall and also have roles during cell separation processes such as those controlling organ abscission (Hadfield et al., 1998; Ogawa et al., 2009; Swain et al., 2011).

The oil palm (*Elaeis guineensis* Jacq.) fruit has two types of AZs, one large multilayer primary AZ and up to four adjacent AZs that are less distinguishable. The primary AZ is in the boundary between the pedicel and mesocarp tissues at the base of the oil palm fruit, while the adjacent AZs are at the periphery of the primary AZ at the base of the outer whorl organs including the rudimentary androecium, tepals, and the bracteole (Henderson and Osborne, 1990, 1994; Henderson et al., 2001). The primary AZ of ripe oil palm fruit has high levels of un-methylesterified pectin proposed to contribute to the spatial specificity of cell separation (Henderson et al., 2001). Separation takes place first in the primary AZ while adjacent less distinguishable AZs separate only after the primary zone has separated, which suggested a signal generated in the primary AZ is necessary for adjacent AZ cells to separate (Henderson and Osborne, 1994). The primary AZ is larger and clearly more visible than the adjacent AZs, which are far less distinguishable. A previous study indicated that the primary AZ arises early during floral development, but more detailed information about the morphogenesis of this exceptionally large primary AZ is lacking (Henderson and Osborne, 1990).

Previous data showed the AZ response to ethylene depends on the stage of fruit development; the ripest fruit AZ responds quickest and function to allow rapid cell separation (Roongsattham et al., 2012). The objectives of this work were to examine in detail the intra- and extra-cellular changes during the development and function of the ripe fruit AZ to provide insight in to the underlying mechanisms. We use histological and immunohistochemical methods combined with a multi-imaging approach including confocal, electron, and Fourier-transformed infrared microscopy to examine the cellular characteristics of the oil palm fruit AZ during development and abscission.

## Materials and methods

### Plant material

Oil palm *E. guineensis* fruits (type *dura* of Deli Dabou origin) from CRA-PP Pobé plantation, Benin were used for classical histology experiments. Oil palm *E. guineensis* fruits (type *tenera* clone C) from Golden Tenera Plantation in Krabi Province, Thailand were used for immunocharacterization experiments. Fruit sampled at different stages of development were selected from bunches as described previously (Roongsattham et al., 2012).

### Light microscopy and image acquisition

Samples were fixed in 0.2 M phosphate buffer containing 2% (w/v) paraformaldehyde, 1% (w/v) caffeine, and 2% (v/v) glutaraldehyde 25% for a minimum of 2 days at 4°C as previously described (Buffard-Morel et al., 1992). Serial dehydration with ethanol (EtOH) from 30 to 100%, then 100% butanol/100% EtOH (v/v), and finally 100% butanol was performed for each sample and followed by impregnation and embedding in Technovit 7100 resin (Heraeus Kulzer). Semi-thin sections of 3 μm were cut using a microtome. Each section was stained with toluidine blue, ruthenium red, alcian blue (AB), AB, and periodic acid-Schiff (ABS). Ruthenium red is a commonly used dye to visualize pectin, which selectively binds to the intramolecular spaces of carboxyl groups of pectin (Sterling, 1970; Hou et al., 1999; Leroux et al., 2007). Toluidine blue is a metachromasia compound that stains lignin and phenols to bluish-green with a pH-independent covalent bonding and non-lignin cell wall components to reddish-violet (acid; e.g., pectin) and bluish-violet (neutral; Conrad, 2008). For ABS, sections were double-stained with Periodic Acid-Schiff (PAS) reagent, combined with AB. A description of the stains used in the study is included in Supplementary Table 1. Photomicrographs were taken with a Leica camera on a Leica (LEITZ DMRB) light microscope (x20/0.5; x40/0.7; and x100/1.3). Samples were taken and observations were made with fruit bases containing the AZ from at least three different fruit collected.

### Electron microscopy

The samples were prepared and analyzed as described previously (Verdeil et al., 2001). Samples taken from the central cell layers in the center of the primary AZ between vascular strand parenchyma cells (1 mm^3^ cubes of tissue) were fixed in a 0.05 M Sorensen buffer solution (pH 7.3) containing 2.5% glutaraldehyde and gently stirred for 16 h at 4°C. Cubes were then rapidly rinsed with distilled water (3 × 10 min), then post-fixed in 1% aqueous osmium tetroxide containing 3% sucrose for 2 h at 20 °C in the dark. They were then dehydrated in an EtOH dilution series (30, 50, 70, and 90% EtOH; 10 min each) and finally for 15 min in pure EtOH; samples were then embedded in Epon EmBed 812 using an Automated Microwave Tissue Processor (Leica EM AMW). Ultrathin sections (thickness around 80 nm) were obtained with a Leica-Reichert Ultracut E ultramicrotome, and then stained with uranyl acetate in EtOH. Sections were then mounted on Ni-grids and examined with a Hitachi 7100 electron microscope.

### Immunohistochemistry

Samples (base of the fruit including the AZ) were fixed in 1X Phosphate-buffered saline (PBS) buffer containing 4% (w/v) paraformaldehyde for a maximum of 16 h at 4°C in dark. After fixation, samples were washed twice in 1X PBS buffer with glycine (2%), then twice in 1X PBS buffer. The samples were then dehydrated in EtOH (incubations in 50% EtOH for 1 and 2 h, then 70% EtOH for 1 and 2 h at RT) and samples were preserved in 70% EtOH at 4°C. Stored tissues were cut at 150 μm thickness with a vibratome (Microm HM 650V, Walldorf, Germany). The sections were placed in glass wells containing PBS solution (pH 7.4), then treated with blocking buffer (BB; 6% bovine serum albumin):PBS = 1:1 (500–1000 μl) and shook gently on a shaker for 3 h at room RT. BB:PBS:primary rat antibody (JIM5, JIM7, LM7, or LM8; Willats et al., 2000, 2004; Clausen et al., 2003) at 9:9:2 was prepared. The PBS-BB solution was removed and replaced with BB:PBS:primary rat antibody. Samples were agitated gently on a shaker overnight (18 h) at 4°C. After incubation, the BB:PBS:primary rat antibody solution was removed and replaced with PBS solution and shook gently on a shaker for 15 min, then repeat this step two times. After this step, all preparations were performed in the dark. Prepare BB:PBS:secondary antibody (Alexa Fluor® 546 Goat Anti-Rat, INVITROGEN, https://www.lifetechnologies.com) at 499:499:2. Alexa 546 was chosen because minimal autofluorescence was found in oil palm fruit tissue corresponding to the windows of spectral emission of this dye. The tissue observation was performed on a Zeiss LSM 510 Meta Confocal Microscope. Microscope imaging was performed at IGH, Montpellier RIO Imaging Platform (www.mri.cnrs.fr) with a multiphoton laser scanning Axiovert 200M 510 META NLO microscope (Carl Zeiss MicroImaging, Jena, Germany) using the multitrack image acquisition. Excitation was at 543 nm with laser power 65% for Alexa 546 and at 405 nm with laser power 7% for DAPI, a fluorescent nuclear stain that binds to A/T rich DNA repeats, with an emission peak of 460 nm (Kapuscinski, 1995). Sections were observed with an immersion objective C-Apochromat 40X/1.2 W and pictures were acquired using the ZEISS LSM image browser software (Carl Zeis MicroImaging GmbH Standort Göttingen—Vertrieb Deutschland, Göttingen, Germany; www.zeiss.com). A description of the antibodies used in the study is included in Supplementary Table 1.

### Cell parameter measurements

Cell parameter measurements were performed for cell width, cell wall width, and middle lamella width by using the ImageJ program (http://rsb.info.nih.gov/ij/). The cell width measurements were performed from the histology samples stained with toluidine blue or ruthenium red. The cell wall and the middle lamella width measurements were performed from the confocal microscope images from immunolocalization studies. The cell parameter data were statistically treated with one way ANOVA analyses and then Post Hoc tests were performed for multiple comparisons with DunnettT3 by using the Statistical Package for the Social Sciences (SPSS) Statistics 17.0 (http://www-01.ibm.com/software/analytics/spss/).

### Fourier-transform infrared (FT-IR) microspectroscopy and degree of esterification measurements

The FT-IR microspectroscopy was performed as described previously using an IN10 Thermo Infrared Imaging system (Mouille et al., 2003). Sections of 50 μm were cut using a vibratome. From the average spectra data for each sample, the degree of methylesterification (DME) was estimated using the formula DME = A1740/(A1740 + A1630) described previously, where absorbance at 1630 cm^−1^ corresponds to the COO^−^ group and absorbance at 1740 corresponds to the carbonyl group from both COOH and COOCH_3_ (Chatjigakis et al., 1998; Manrique and Lajolo, 2002; Gribaa et al., 2013). A total of 7–11 replicates for each sample were analyzed by FT-IR and used to quantify the DME. The A1630-value was obtained by the average between A1628 and A1632. Statistical significance of the DME between sample groups was calculated by ANOVA at *P* = 0.01.

## Results

### Distinct developmental characters accompany the capacity for AZ cells to function in ripe fruit

**Figure 1:**
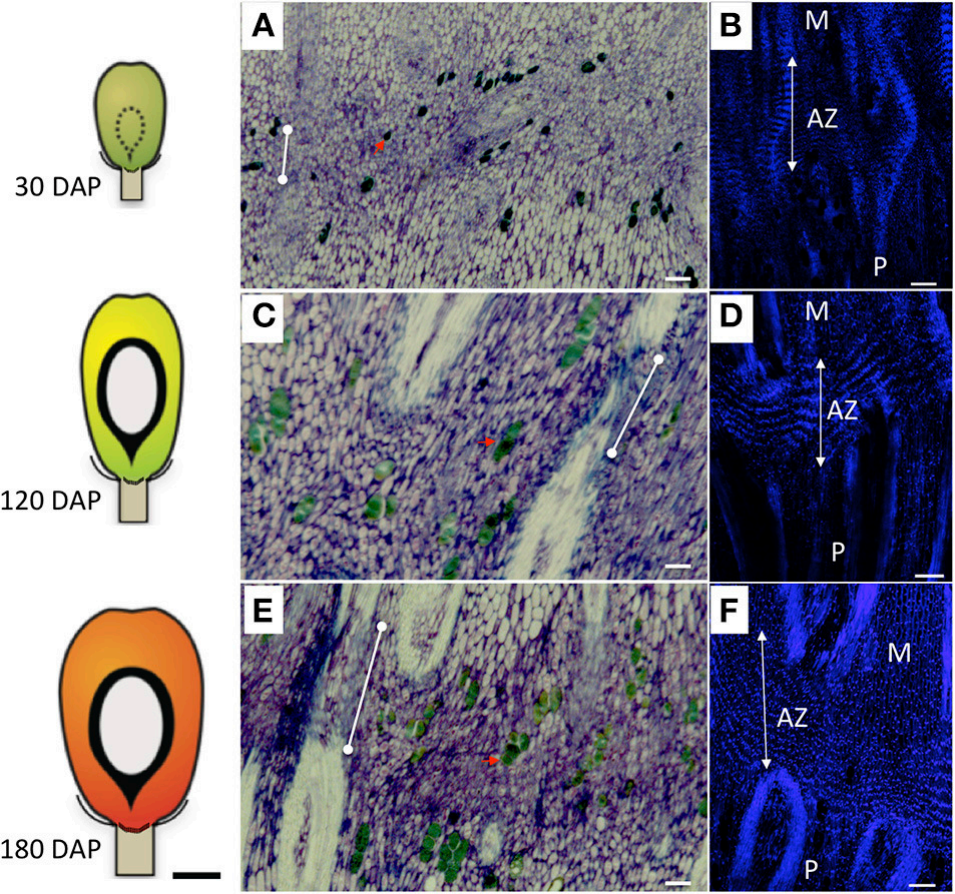
**Development of the AZ in the base of the oil palm fruit**. Longitudinal sections of oil palm fruit bases at 30 days after pollination (DAP, **A,B**), 120 DAP **(C,D)**, and 180 DAP **(E,F)** were stained with toluidine blue **(A–C)** or DAPI **(B,D,F)**. Schematic fruit diagrams to the left of photos correspond to the stages of development at which time the fruit tissues were collected (Tranbarger et al., 2011). The primary abscission zone (AZ) is depicted with dotted lines positioned between the mesocarp (M), and pedicel (P); red arrows, phenolic containing cells; dots connected by white lines, undifferentiated constricted vascular tissue; white lines with double arrows, nuclear alignment within AZ cell layers; Scale bars for photos are 100 μm; Scale bar for schematic fruit diagrams is 10 mm.

In this study, we examined in detail the morphogenesis of the primary AZ compared to adjacent pedicel and mesocarp tissues. The results presented here are based on observations of at least three or more samples and the photos were selected to represent the most consistent features observed. Longitudinal sections through the base of the fruit were made to examine the cellular characteristics of the primary AZ compared with the adjacent mesocarp and pedicel tissues (Figures 1A–F). The primary AZ was easily observed at 30 days after pollination (DAP) in fruit sections stained with toluidine blue and consisted of small densely cytoplasmic cells as previously described (Figure 1A; Henderson and Osborne, 1990). At 30 DAP, vascular parenchyma tissues were not lignified in any of the tissues (Figure 1A). By 120 DAP, vascular bundles were differentiated and lignified in the pedicel and mesocarp, while lignification was interrupted in the AZ vascular bundles at both 120 and 180 DAP (Figures 1C,E, dots connected by white lines). As with a previous study, phenolic containing cells were observed near the primary AZ, but unlike the previous study, these specialized cells were also located within the primary AZ layers (Figures 1A,C,E, red arrows; Henderson and Osborne, 1990). The polyphenol-containing cells appeared larger by 120 DAP and were often found grouped in two or more cells (Figures 1C,E).

**Figure 2:**
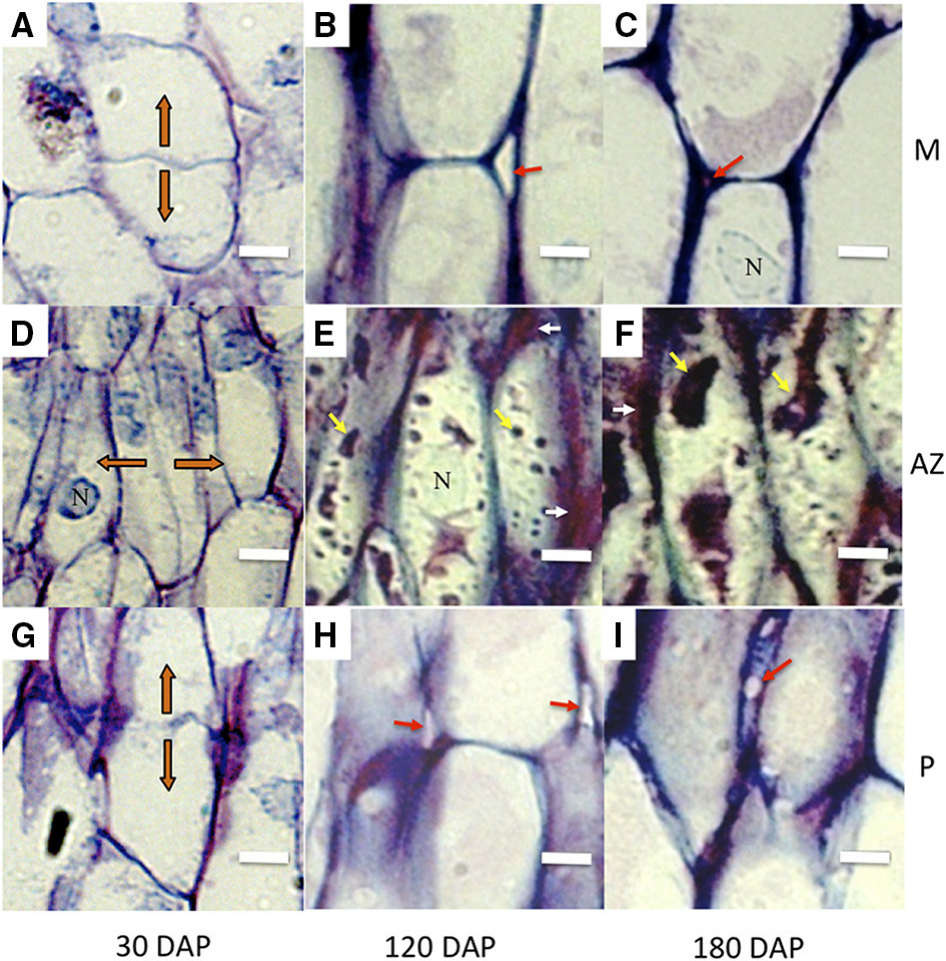
**AZ cells have distinct characteristics during development compared to adjacent mesocarp and pedicel cells**. Longitudinal sections of the oil palm fruit bases were stained with toluidine blue. **(A–C)**, mesocarp (M) cells, **(D–F)**, AZ cells, **(G–I)**, pedicel (P) cells. Orange arrows, orientation of recently divided cells; white arrows, tightly packed tip regions of AZ cells; red arrows, intercellular space openings; yellow arrows, intracellular material; DAP, days after pollination; N, nucleus; Scale bars are 10 μm.

Longitudinal sections of 30 DAP fruit stained with DAPI revealed that the nuclei of the AZ layer cells were aligned, particularly in the developing vascular bundles at this stage, then became more visibly aligned throughout the primary AZ during development at 120 and 180 DAP (Figures 1B,D,F). At 30 DAP, recently divided cells in the mesocarp, AZ and pedicel tissues were observed (Figures 2A,D,G). In the AZ, the plane of recent cell division was periclinal (parallel to the outer fruit surface), while in the mesocarp and pedicel cell divisions were anticlinal (plane perpendicular to the outer fruit surface). At 120 DAP cell divisions were no longer evident, and cells appear elongated in all tissues, and AZ cells appeared narrower than mesocarp and pedicel cells (Figures 2B,E,H).

**Figure 3:**
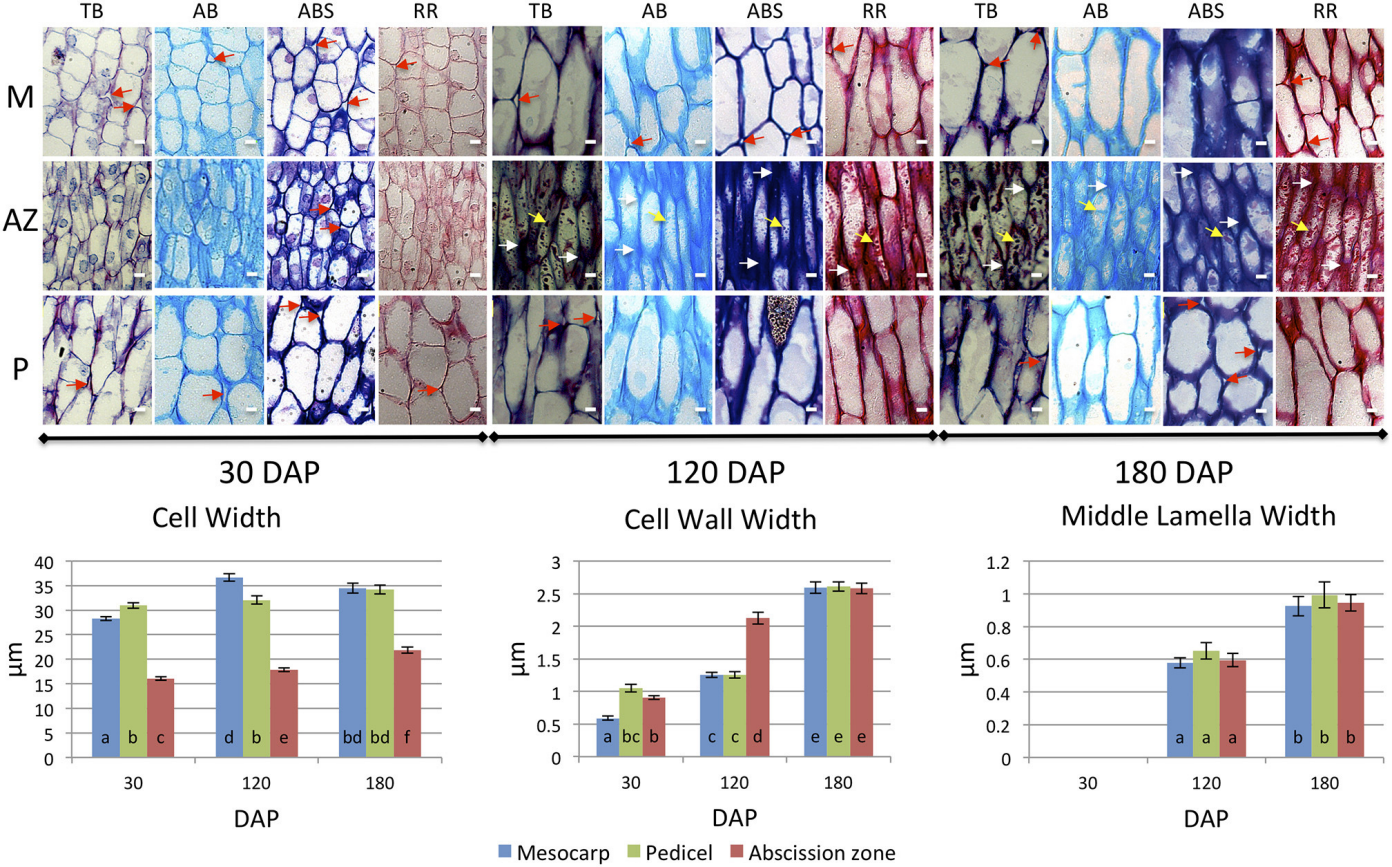
**Development of mesocarp (M), AZ and pedicel (P) cells compared provides evidence for differences in cell wall assembly**. Upper images, longitudinal sections of the oil palm fruit base were stained with toluidine blue (TB), alcian blue (AB), alcian blue and periodic acid-Schiff (ABS), or ruthenium red (RR) as indicated. White arrows, cell wall thickenings at tips of adjacent AZ cells; red arrows, intercellular space openings; yellow arrows, intracellular material; DAP, days after pollination; Scale bars are 10 μm. Graphs below: measurements of M, AZ and P cell, cell wall, and middle lamella widths during development. Different lower case letters represent statistically significant differences. The error bars represent standard error.

During development, AZ cells had dark staining intercellular spaces often located to the tips of cells suggesting concentrated areas of secretion related to cell wall biosynthesis activity (Figures 2E,F, 3). In contrast, mesocarp and pedicel cells had intercellular spaces that stain less intensively and appeared often to become regions of detachment that led to airspace formation (Willats et al., 2001b; Jarvis et al., 2003). Intercellular airspaces were rarely observed in the AZ cell layers (Figure 3). Also during development, dark staining particles in AZ cells appeared to be localized in the cytoplasm that became evident during ripening at 120 and 180 DAP. These intracellular particles appeared to contain pectin, based on coloration with both toluidine blue (Figures 2E,F) and ruthenium red staining (Figure 3). Similarly stained particles were not observed in either the mesocarp or pedicel cells at 120 or 180 DAP (Figures 2B,C,H,I, 3).

To complement this comparative study of the pedicel, AZ and mesocarp tissues, we measured several quantitative parameters of the cells including: cell width, cell wall width, and middle lamella width. The cell width measurement data from longitudinal sections revealed that at 30 DAP, the average width of a mesocarp cell was 28.29 μm and significantly increased to 36.67 μm at 120 DAP. In contrast, the average pedicel cell width was 30.94 μm at 30 DAP and did not significantly increase at 120 or 180 DAP while in the AZ, the average cell width was 16.05 μm at 30 DAP and significantly increased to 17.84 μm at 120 DAP and to a maximum of 21.87 μm at 180 DAP. Notably, the average AZ cell width was less than the other tissues at all developmental stages examined (Figure 3, Supplementary Tables 2–5).

For the cell wall, the average mesocarp cell wall width was 0.59 μm at 30 DAP and significantly increased to 1.25 μm at 120 DAP and 2.59 μm at 180 DAP. In contrast, the average pedicel cell wall width was 1.05 μm at 30 DAP, did not change significantly at 120 DAP, but increased to 2.61 μm at 180 DAP. In contrast to these two adjacent tissues, the average AZ cell wall width was 0.91 μm at 30 DAP and significantly increased to 2.12 μm at 120 DAP, thicker than both the mesocarp and pedicel cells, which suggests more rapid cell wall biosynthesis occurs in the AZ between 30 and 120 DAP than the adjacent tissues. At 180 DAP, the AZ cell wall width was at a maximum of 2.58 μm, while all tissues display the maximum width and there was no statistically significant difference between the tissues at this stage (Figure 3, Supplementary Tables 6–9).

At 30 DAP the middle lamella width was too small to measure by our method at this early stage of development. At 120 DAP, the average mesocarp cell middle lamella width was 0.58 μm and significantly increased to 0.93 μm by 180 DAP. In the AZ, the average middle lamella width was 0.59 μm at 120 DAP and significantly increased to 0.94 μm by 180 DAP. In the pedicel, the average middle lamella width was 0.65 μm at 120 DAP and significantly increased to 0.99 μm by 180 DAP. Interestingly, the middle lamella width of each cell type was similar at each stage of development, and increased similarly between 120 and 180 DAP (Figure 3, Supplementary Tables 10–13).

**Figure 4:**
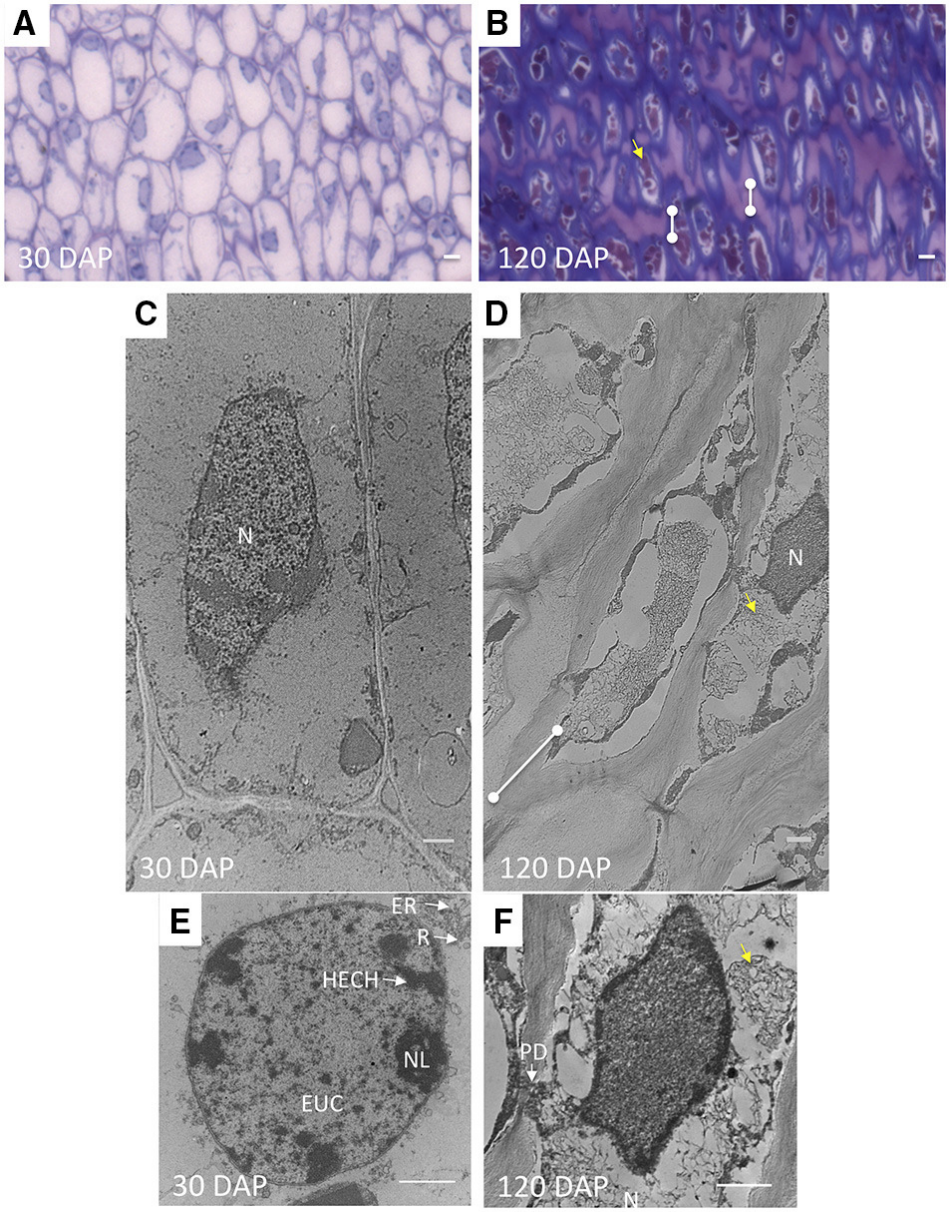
**The shape and characteristics of AZ cells and nuclei change during development related to the acquisition of abscission competence. (A,C,E)** longitudinal ultra-thin sections of AZ cell layers at 30 DAP and **(B,D,F)** 120 DAP prepared for transmission electron microscopy **(C–F)**, semi-thin longitudinal section of the same samples stained with toluidine blue **(A–B)**. N, nucleus; HECH, heterochromatin; EUC, euchromatin; NL, nucleolus; R, ribosome; ER, rough endoplasmic reticulum (RER); dots connected by white lines, cell wall expansion at cell tips; yellow arrows, fibrous matrix or intracellular particles. Scale bars in **(A)** and **(B)** are 10 μm; Scale bars in **(C–F)** are 1 μm.

**Figure 5:**
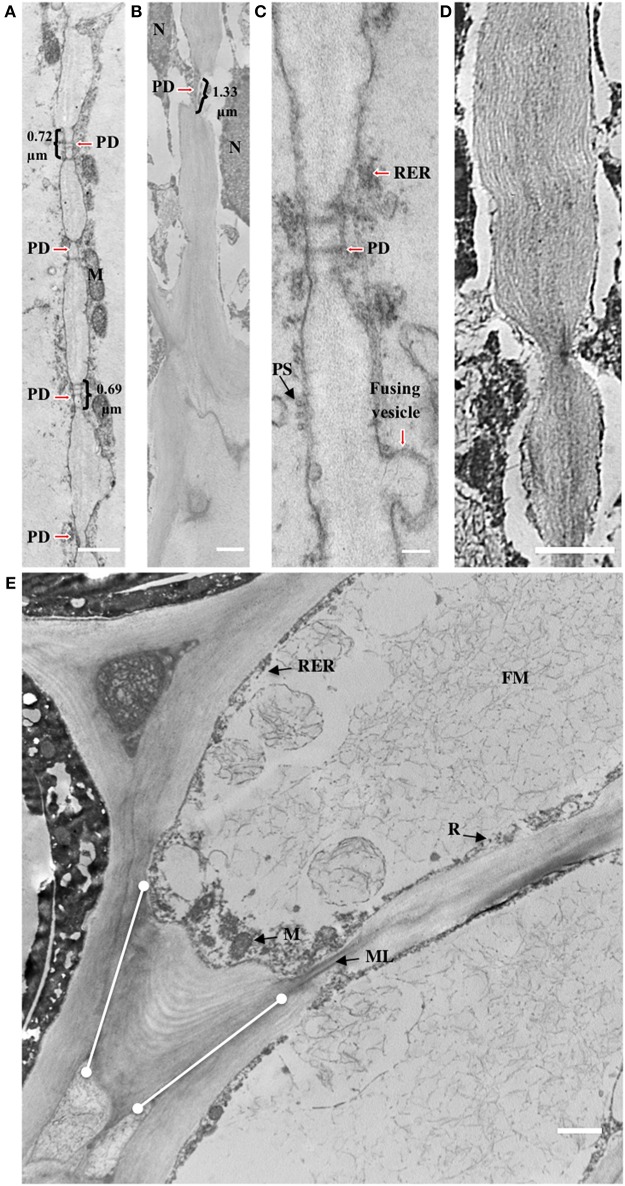
**Cell wall striations appear and plasmodesmata increase in size during development of the AZ layer cells. (A,C)** 30 DAP; **(B,D,E)** 120 DAP; PD, plasmodesmata; R, ribosome; M, mitochondria; ML, middle lamella; FM, fibrous matrix material; PS, polysome; dots connected by white lines, cell wall expansion layers at cell tips. Scale bars in **(A–E)** are 1 μm and 0.1 μm in C.

To examine the ultrastructure changes that occur during AZ morphogenesis, electron microscopy was performed at 30 and 120 DAP. Thin longitudinal section preparations for electron microscopy analysis of the AZ of 30 and 120 DAP fruit stained reddish-violet with toluidine blue corroborating the presence of pectin material within AZ cells, and an increase cell wall width apparently rich in pectin, particularly at the tips of cells between AZ layers (Figures 4A,B). Electron microscopy analysis revealed a dramatic increase in cell wall width that occurs between 30 and 120 DAP, which corroborates the increase in cell wall width measured (Figures 3, 4A–D, 5A–D).

Accompanying these changes, AZ cell shape at 30 and 120 DAP cells were rectangular but more elongated at 120 DAP (Figures 4C,D). The AZ cell nuclei at 30 DAP were rounded or slightly elliptical, while those at 120 DAP were more amoeboid shaped (Figures 4E,F). Nucleoli could be observed at 30 DAP but were rarely found in 120 DAP (Figures 4E,F). At 30 DAP, nuclei with highly condensed chromatin were observed (Figure 4E). Interestingly, at the tip of the elongated AZ parenchyma cells, the cell wall was thickened with the appearance of multiple striated layers or ripples that could be either continuous with the middle lamella or the primary cell wall (Figure 5E). Finally, ribosomes, rough endoplasmic reticulum, mitochondria, and vesicles were observed at the periphery of the cytoplasm, in particularly at the tip of the cells (Figure 5E), and a fibrous matrix can be found throughout the cell interior (Figures 4D,F, 5E). Taken together, these ultrastructural features suggest extensive cellular activity in the AZ elongated cell tips including high protein synthesis and metabolic activity possibly related to exocytosis.

Plasmodesmata (PD) in the cell walls at 30 DAP between cells in the same AZ layer (intra-layer PD) were frequently observed, but less frequent between cells in adjacent AZ layers (inter-layer PD, Figures 5A,C). PD appear to be grouped together in areas of cell wall constriction which became more pronounced by 120 DAP (Figures 5A–D). At 120 DAP, PD appeared less frequent but the constricted cell wall regions where PD were localized increased in width and were situated in the middle of the cell between cells of the same layer. The constricted regions with PD increased almost twofold between 30 and 120 DAP (Figures 5A–D). Nuclei were often closely associated to PD, while vesicle fusion to the plasma membrane could be observed adjacent to thickened cell walls at 120 DAP (Figure 5C).

### Cellular changes occur in the AZ cells of ripe fruit during abscission

**Figure 6:**
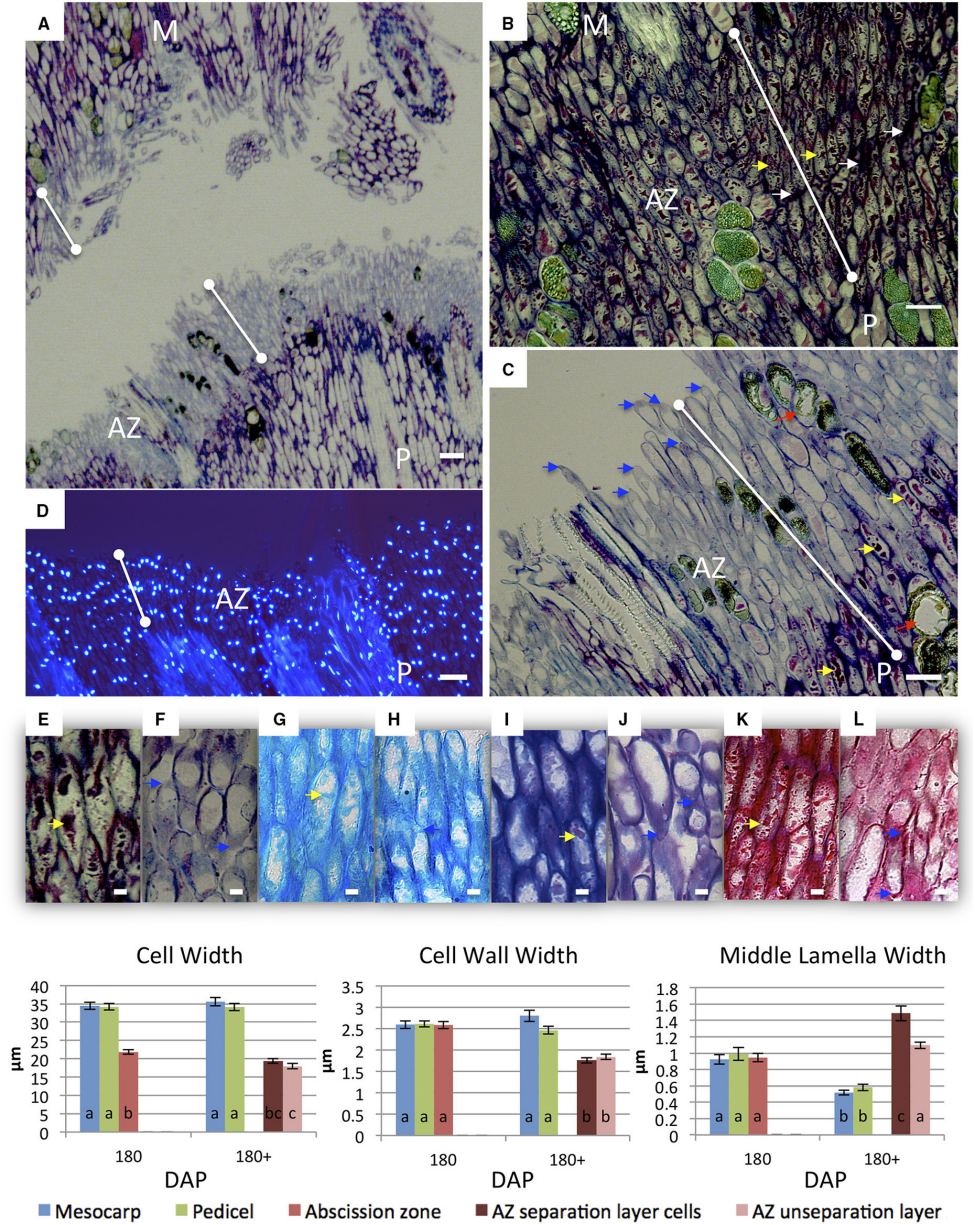
**Changes in AZ cells of ripe fruit occur during cell separation induced by ethylene treatment**. Longitudinal sections of the oil palm fruit before **(B,E,G,I,K)** and after 9 h of ethylene treatment **(A,C,D,F,H,J,L)** cell separation in the AZ stained with toluidine blue **(A–C,E,F)**, DAPI **(D)** alcian blue **(G,H)**, alcian blue and periodic acid-Schiff **(I,J)**, or ruthenium red **(K,L)**. AZ before and after cell separation; red arrows, degraded phenolic containing cells; white arrows, intercellular space thickenings; yellow arrows, intracellular material; blue arrows, tip elongation with evidence of secretion adjacent separation sites; dots connected by white lines, abscission zone (AZ); M, mesocarp; P, pedicel; Scale bars **(A–D)** are 100 μm; Scale bars **(E–L)** are 10 μm. Graphs below: measurements of M, AZ and P cell, cell wall, and middle lamella widths before and after cell separation in the AZ. Different lower case letters represent statistically significant differences. The error bars represent standard error.

To assess the intra- and extra-cellular changes that occur in the AZ during abscission, we examined ripe fruit AZ cells before and after cell separation induced by ethylene (Roongsattham et al., 2012). Cell separation was commonly observed within AZ cell layers and not between the AZ layers and adjacent mesocarp or pedicel tissues (Figures 6A,C). In the example presented, cell separation occurred closest to the mesocarp, but this was variable and not always the case; separation can also be in the middle of the AZ layers or closer to the pedicel tissue, but was not observed between AZ/pedicel or AZ/mesocarp cells.

During and after cell separation occurs, no evidence for cell rupture or cell division was observed (Figure 6). One prominent characteristic after cell separation was the change of toluidine blue staining from reddish-violet to bluish violet in the AZ cell walls, which suggests a change of cationic environment (i.e., Ca^2+^ and/or H^+^) in this region that could be related to pectin modification in the AZ (Figures 6A–C,E,F). In contrast, pedicel and mesocarp cell walls stained dark with toluidine blue even after separation in the AZ occurred (Figures 6A–C). After separation in the AZ, the nuclei remained aligned and intact and there is no visible evidence of cell death based on the integrity of the nuclei (Figure 6D). Another prominent characteristic of AZ cells after separation was the change in the intracellular particles that accumulated in the AZ cells during development (Figures 2D–F, 3, 6A–C,E–L). Once separation occurred, the AZ cells appeared to have fewer particles as visualized by a decreased coloration with different histochemical stains (Figures 6A,C,E–L). Finally, cells in the AZ layers appeared to undergo tip elongation and there was evidence of exocytosis adjacent to separation sites (Figures 6F,H,J,L).

We then measured the changes in cell, cell wall and middle lamella widths after ethylene induced cell separation in 180 DAP fruit (Figure 6). The average cell width of mesocarp and pedicel cells did not significantly change in 180 DAP fruit after ethylene treatment. In contrast, the average cell width of AZ layer cells before ethylene treatment (21.87 μm) decreased significantly in unseparated AZ cells (17.99 μm), and non-significantly in cells that separated (Figure 6, Supplementary Tables 14–17). The average mesocarp and pedicel cell wall width at 180 DAP (2.59 and 2.61 μm, respectively) did not change significantly after ethylene treatment. In contrast, the average cell wall width (2.58 μm) decreased significantly after ethylene treatment in both the separation layer and unseparated AZ layer cells (1.76 and 1.84 μm, respectively, Supplementary Tables 18–21). Finally, the average middle lamella width of mesocarp cells at 180 DAP (0.93 μm) decreased significantly (0.52 μm) after ethylene treatment. A similar decrease was observed in pedicel cells; the average width of the middle lamella was 0.99 μm and significantly decreased to 0.58 μm after ethylene treatment. In contrast, the average middle lamella width of the AZ cells was 0.94 μm, and significantly increased after ethylene treatment to 1.49 μm in separated layer cells, while insignificantly increased to 1.10 μm in cells of the unseparated layers (Figure 6, Supplementary Tables 22–25). The increase in the AZ cell middle lamella width suggests this area is undergoing expansion prior to cell separation. The results reveal distinct and dynamic changes occur in the AZ cells in association with the cell separation and abscission process in contrast to adjacent mesocarp and pedicel tissues.

### FT-IR microspectroscopy reveal changes in methylesterification status occur in AZ cell walls during cell separation

**Figure 7:**
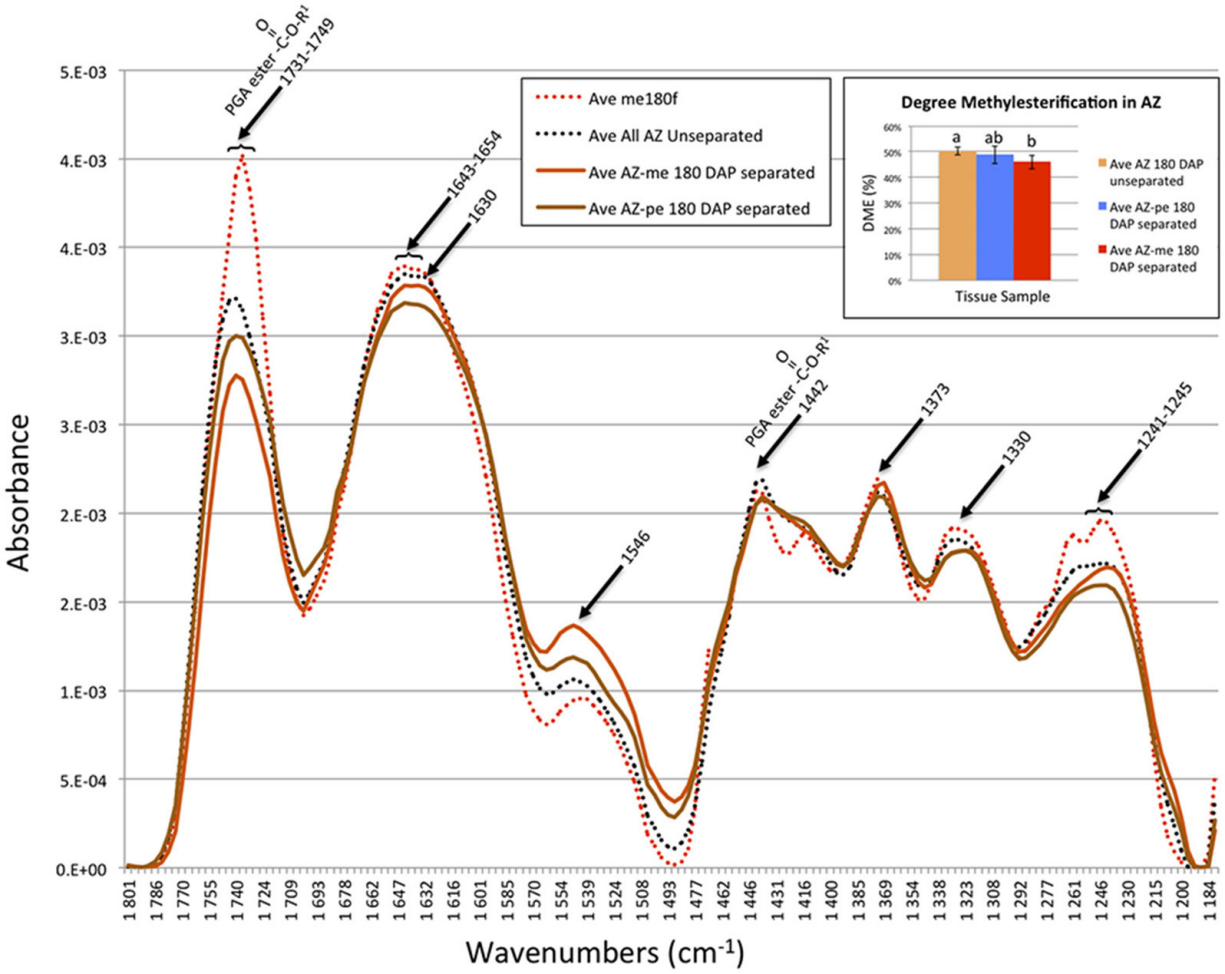
**FT-IR microscopy spectra suggest a decrease in the degree of pectin methylesterification (DME) occurs during cell separation in AZ cells closest to the mesocarp**. Ave, average absorbance from 11–29 replicates; PGA, polygalacturonic acid; me, mesocarp cell; AZ-me, separated cells in the AZ closest to the mesocarp; AZ-pe, separated cells in the AZ closest to the pedicel. Graph inset, the degree of pectin methylesterification (A_1740_/[A_1740_ + A_1630_]) in the oil palm AZ cells during separation calculated from the FT-IR spectroscopy data (see Section Materials and Methods). Error bars indicate standard error. Mean values followed by different letters are statistically different.

To examine specific changes that occur in the AZ cell walls during cell separation in more detail, FT-IR microscopy was used to compare unseparated and separated AZ cells (Figure 7). The spectra revealed that mesocarp cells had higher absorbance at 1731–1749 (a range that corresponds to both the COOH and methylester carbonyl group) than either separated or unseparated AZ cells, which suggested a higher esterification in mesocarp cells (Chatjigakis et al., 1998; Manrique and Lajolo, 2002; Gribaa et al., 2013). Furthermore, unseparated AZ cells had a higher absorbance at 1731–1749 than separated cells in both the distal (close to mesocarp) or proximal (close to pedicel) AZ separation layer cells, which suggested a higher esterification in unseparated cells. From the spectra data, the DME was estimated (see Material and Methods for description) that revealed a significant decrease in DME occurs within separated AZ cells in the distal layers after separation (Figure 7, Graph inset).

### The Jim5 epitope signal increases prior to cell separation and is high at cell edges and cells that have undergone separation

The classical histology, electron microscopy, and cell parameter analyses described above provide evidence for changes in the cell wall of AZ layer cells during ethylene-induced abscission, in particular changes related to pectin. We used the antibodies JIM5, JIM7, and LM7 to distinguish the cellular localization of partially methylesterified HG, and LM8 to recognize xylogalacturonan (XGA), another form of pectin that is associated with detached cells (Knox et al., 1990; Clausen et al., 2004; Willats et al., 2004). For each experiment, the fluorescent nuclear stain DAPI was used to ensure the region examined was the AZ by the observation of the nuclear alignment of the AZ layer cells (Figures 1B,D,F). The immunohistological controls included incubation without primary antibodies, with secondary antibodies with the different tissues examined (Supplementary Figure 1).

**Figure 8:**
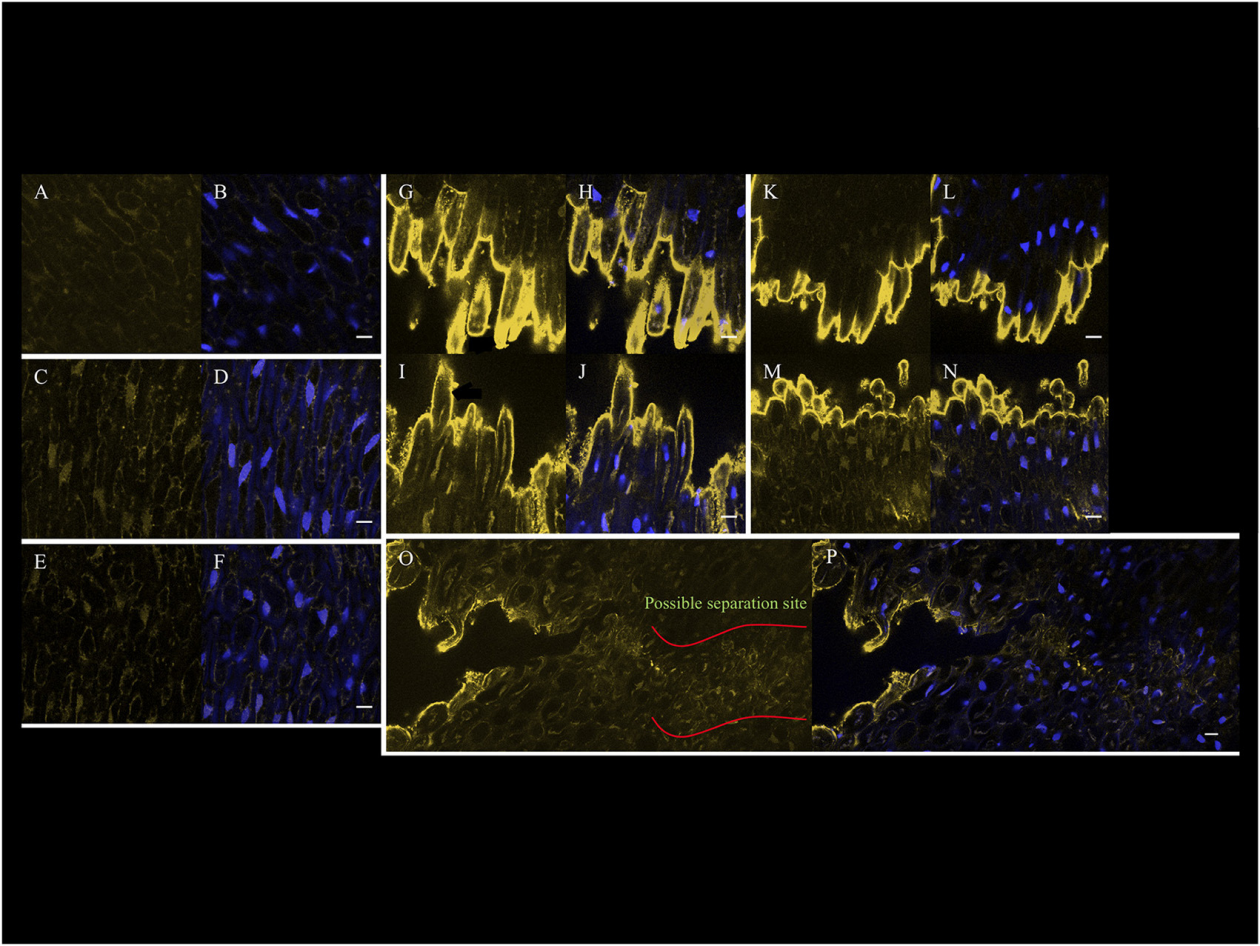
**Confocal immunohistological images of the JIM5 epitope signal that increases in the AZ cells prior to and after cell separation on the surfaces of separated cells of 180 DAP fruit. (A)** JIM5, and **(B)** JIM5 and DAPI AZ 3 h without ethylene treatment. **(C)** JIM5, and **(D)** JIM5 and DAPI AZ 3 h with ethylene treatment. **(E)** JIM5, and **(F)** JIM5 and DAPI AZ 6 h with ethylene treatment. **(G)** JIM5, and **(H)** JIM5 and DAPI AZ at M side at 9 h with ethylene treatment and separation occurring. **(I)** JIM5, and **(J)** JIM5 and DAPI AZ at P side at 9 h with ethylene treatment and separation occurring. **(K)** JIM5, and **(L)** JIM5 and DAPI AZ at M side at 12 h with ethylene treatment and separation occurring. **(M)** JIM5, and **(N)** JIM5 and DAPI AZ at P side at 12 h with ethylene treatment and separation occurring. **(O)** JIM5, and **(P)** JIM5 and DAPI of samples where both separated and non-separated cells can be observed at 9 h with ethylene treatment. Scale bars are 10 μm.

The signal intensity that corresponds to the JIM5 epitope in the AZ from ripe fruit (180 DAP) after 3 h with air treatment was very low (Figures 8A,B). In contrast, by 3 and 6 h after ethylene treatment, the signal obtained with JIM5 increased on the inner side of primary cell wall (Figures 8C–F). By 9 h after ethylene treatment when separation had occurred, the signal increased dramatically at sites of separation of AZ cells on both the mesocarp (distal) and pedicel (proximal) sides (Figures 8G–J). Signals on the inner side of the primary cell wall and in cytoplasm could be detected, in addition to line like signals that arise from deeper within the AZ cells and/or cell layers. A film was made to give a three-dimensional overview of the JIM5 signal in AZ after separation at 9 h (Supplementary Movie 1). The film allows visualization of the high JIM5 signal along the separated cell surfaces, in addition to the lower signals deeper in the AZ layers where cells have not yet separated. By 12 h after ethylene treatment, the signal at the separated primary cell wall of separation layer cells of both the mesocarp and pedicel sides remained high, while the line like signals were lost from the mesocarp side (Figures 8K–N). In samples where both separated and non-separated cells could be observed at 9 h with ethylene treatment, the signal was not only observed on the exposed separated cell surfaces, but also appeared to increase in non-separated cells which may indicate the future separation sites (Figures 8O,P). In addition, the signal intensity was seen all along the separated cell layer surface (Supplementary Figure 2). Moreover, the signals at possible future separation sites within the non-separated zone were quantified using the profile option of the Zeiss LSM image browser software and the results suggested that indeed the JIM5 epitope increased in certain AZ cells prior to separation, and may predict the future sites of cell separation (Supplementary Figure 3).

No JIM7 epitope signal was detected in the AZ of 180 DAP fruit at 3 h without ethylene treatment or at 3 and 6 h with ethylene treatment (Supplementary Figures 4A–F). By contrast, at 9 h with ethylene treatment and with separation occurring, the signal increased at the separated primary cell wall of separation layer cells on both mesocarp and pedicel side (Supplementary Figures 4G–J). Similar to the signal at 9 h, the signal at 12 h with ethylene treatment at the separated primary cell wall of separation layer cells in both mesocarp and pedicel side of AZ remained high (Supplementary Figures 4K–N). However, the signal interior to the separation face decreased on the mesocarp side while the signal on the pedicel side remained high. In contrast to the JIM5 epitope, the JIM7 epitope did not reveal an increase in cells prior to separation, only in separated cells (Supplementary Figures 2, 4O,P).

The signal corresponding to the LM7 epitope in the AZ of all samples (3 h without ethylene treatment, 3, 6, 9, 12 h with ethylene treatment) was very low and not associated with cell separation (Supplementary Figures 5A–N).

The signal corresponding to the LM8 epitope after 3 h without ethylene treatment was undetectable but becomes stronger after 9 and 12 h of ethylene treatment and only after AZ cell separation (Supplementary Figures 6A–N). By contrast, the pattern of the signal observed was spotted and appeared to be associated to the vascular tissue cells (Supplementary Figures 2I–L, 6G–N). In comparison, the JIM5 and JIM7 epitopes were observed all along the separation surfaces on both the mesocarp and pedicel sides of the AZ (Supplementary Figures 2A–H). Notably, the JIM5 epitope appeared to increase earlier than either the JIM7 or the LM8.

## Discussion

### AZ specific regulation during development targets cell wall biogenesis and results in anatomy specialized for intra-AZ-layer cell–cell communication

The duration of development from the early to ripe stages at which time the fruit abscise and are shed from bunches is variable and depends on the genetic material (Fooyontphanich et al., in press). In the current studies, the final stages of ripening ranged from 160 to 190 DAP when the fruit could be observed to detach naturally in the field. During ripening, ethylene production begins to increase by 120 DAP in the mesocarp, and is thought to be the signal that initiates and regulates oil palm fruit abscission (Henderson and Osborne, 1994; Tranbarger et al., 2011). Indeed, several studies have shown that ethylene induces cell separation in the oil palm fruit AZ, while the response to ethylene is developmentally dependent; ripe fruit AZ have the highest capacity to respond to ethylene (Henderson and Osborne, 1994; Roongsattham et al., 2012). A primary objective of this study was to identify cellular characters of the oil palm ripe fruit AZ that correspond to the capacity to respond and function in response to ethylene. During oil palm AZ development, we observed several cellular features that arise during development with timing that corresponds with the capacity for AZ function and response to ethylene.

During early fruit development, the cell division plane in the AZ layer cells is opposite to that observed in both adjacent tissues; periclinal (parallel to the outer fruit surface) in the AZ, and anticlinal in both the pedicel and mesocarp cells. This suggests that AZ expansion is under distinct regulation compared with adjacent tissues from an early stage. Consequently, periclinal cell divisions in the AZ may give rise to the unusual alignment of nuclei between adjacent cells within each AZ layer. Nuclear alignment appears to originate early during vascular tissue differentiation in the base of the fruit then extends across the primary AZ during development. To the best of our knowledge, this is the first observation of nuclear alignment in an AZ. In contrast, no nuclear alignment is observed in the tomato pedicel AZ where cells appear to undergo cell death during the abscission process (Bar-Dror et al., 2011). In the oil palm fruit AZ, our analysis found no evidence for nuclear degradation well after cell separation took place. Interestingly the AZ cell nuclei are closely associated with PD, which could provide an efficient intra-AZ-layer structural capacity to communicate signals and coordinate transcriptional responses, analogous to the observed role for PD in defense signaling (Sager and Lee, 2014; Lee, 2015). Indeed, the timing of PD structural changes between 30 and 120 DAP corresponds to the period when AZ cells acquire the capacity to respond to abscission signals, and may provide the structural anatomical basis to coordinate signals that promote a rapid intra-AZ-layer transcriptional response that leads to cell separation.

The observations that cell wall constrictions are associated with groups of PD between adjacent cells, and vascular cell walls are less lignified within AZ layers, suggests that cell wall biosynthesis and differentiation are under specific control in AZ cells. Indeed, we also show cell wall expansion occurs more rapidly in the AZ than in adjacent mesocarp and pedicel cells, and that this expansion occurs in a polarized manner at the tips of AZ cells (Figures 3, 5). Importantly, the increase in cell wall biosynthesis between 30 and 120 DAP corresponds to the period during which AZ acquire the capacity to respond to ethylene (Roongsattham et al., 2012). Overall, these observations indicate that the control of AZ cell wall biosynthesis is coordinated differentially compared with adjacent tissues, which may be related to the spatial capacity of AZ cells to separate. Differential cell wall construction in the AZ associated with PD development may provide a structural basis for strong intra-layer cell–cell communication required to coordinate abscission signals, while differential cell wall expansion between cell layers may provide the appropriate apoplastic environment for cell separation to take place, analogous to the cell separation that occurs during intercellular space formation (Willats et al., 2001b; Jarvis et al., 2003). Finally, less lignified and differentiated vascular cells in the AZ makes them more susceptible to pectin dissolution by PG. Taken together, AZ cell layer differentiation is controlled by distinct regulation that gives rise to cellular characters that may be important for the capacity to undergo cell separation.

The oil palm AZ consists of a very large primary multi-cell layer AZ that forms a boundary between two fruit tissues with distinct functions; the pedicel for support of the fruit and vascular connection to the bunch, and the mesocarp which has an unusually high capacity for lipid biosynthesis and storage (Tranbarger et al., 2011). As discussed above, we provide evidence that the AZ is under distinct development controls compared to adjacent tissues. Indeed it is known from dicot model species that AZ development and function are highly regulated processes (Estornell et al., 2013). Arabidopsis, boundary AZs are under the control of specific transcription factors including BLADE-ON-PETIOLE1/2 (BOP1/2), ASYMMETRIC LEAVES1 (AS1), and HAWAIIAN SKIRT (HWS) which determine the differentiation, placement and function of floral organ AZs (Gonzalez-Carranza et al., 2007; McKim et al., 2008; Gubert et al., 2014). In the tomato, flower and fruit pedicel AZ differentiation is controlled by a completely different basis including a complex of MADS-box transcription factors (Butler, 1936; Mao et al., 2000; Nakano et al., 2012; Ito and Nakano, 2015). While the molecular basis for these AZ types may be different, it is clear that in both cases the development and function of the AZ is tightly linked; disruption of development affects the capacity to function in the promotion of cell separation and ultimately organ abscission. The current study highlights that strong spatial regulation leads to differential cell wall biosynthesis and assembly processes (e.g., inhibition of the cell wall lignification of vascular bundles across the AZ and extensive cell wall biogenesis at the tips of AZ cells) in the oil palm ripe fruit AZ. Given that the oil palm fruit AZ is multi-cell layered and very large compared to other plant AZs, it provides an interesting experimental system to examine in detail the cellular and biochemical processes that take place during cell separation and abscission, in particular for a monocot fruit species.

### Pectin biosynthesis, metabolism, esterification status, and other cell wall parameters in the oil palm AZ are regulated differently than adjacent tissues

Previous studies revealed that oil palm fruit AZ cell walls are rich in unmethylated pectin and that PG activity and the *EgPG4* transcript is highly expressed in the AZ in response to ethylene (Henderson and Osborne, 1994; Henderson et al., 2001; Roongsattham et al., 2012). These features were proposed as a mechanism to specify cell separation to the AZ in the oil palm fruit base. In the current study, we provide several lines of evidence for the importance of pectin and pectin methylesterification status for the abscission process, and provide a link between ethylene and effect on the AZ cell walls. Firstly, during AZ development, we observed an increase in intra-cellular particles that stained strongly for pectin with ruthenium red. Furthermore, these particles decreased in separated AZ cells after abscission, suggesting a functional importance for the abscission process. Secondly, we observed an increase in the JIM5 epitope by 3 h after ethylene treatment localized to cells in the AZ region before separation. Once separation occurs, the JIM5 epitope increases dramatically on both proximal and distal AZ layer cell surfaces that have undergone cell separation. A previous study described nodular structures thought to be pectin material that appear on the separated cell surfaces (Henderson and Osborne, 1990). The current study provides further evidence that the material that accumulates on the separated cell surfaces is pectin. JIM5 binds strongly to HG, the most abundant pectin polymer of galacturonic acid, with a relatively low methylesterfication status and binds weakly to unesterfied oligogalacturonides (Willats et al., 2000; Clausen et al., 2003). In parallel experiments, we did not see the same pattern of epitope signal with JIM7, which only increased after cell separation. JIM7 recognizes higher order methylesterified HG than JIM5 (Willats et al., 2000; Clausen et al., 2003). In addition, the absence of an increase in the LM7 signal rules out the low methylesterified epitope (four contiguous unesterified galacturonic acid residues adjacent to or flanked by residues with methylester groups) recognized by both JIM5 and LM7, which leaves the moderately methylesterified epitope (three contiguous unesterified galacturonic acid residues adjacent to or flanked by residues with methylester groups) or unesterfied HG as the main epitopes detected by JIM5 in AZ cells prior to and after cell separation. Finally, the LM8 epitope consists of a region of highly substituted xylose in xyloglacturonan (XGA) that is observed in regions of cell separation and complete cell detachment (Willats et al., 2004). In the current study, the LM8 epitope does not increase in cells prior to separation, but is mainly localized to vascular strand cells that have separated, suggesting changes to xyloglacturonan are important for these cell types to separate in the oil palm AZ.

Several studies have examined the cell wall structure before, during and after cell separation in different species undergoing various organ abscission processes (Uheda and Nakamura, 2000; Lee et al., 2008; Bowling and Vaughn, 2011; Iwai et al., 2013). During *Azolla* branch abscission, the JIM5 epitope signal disappeared in the fracture surface of the separated cell (Uheda and Nakamura, 2000). Similarly, during impatiens leaf abscission, a loss of the JIM5 (unmethylesterfied HG) epitope signal was observed in the cell wall/middle lamella at the face of the separation surface of separated cells (Bowling and Vaughn, 2011). In contrast, during the induction of AZ differentiation in the poinsettia leaf base, a demethylesterification of HG was inferred by the differential signals observed with JIM5 (high signal for unmethylesterified HG) and JIM7 (low signal for partially methylesterified HG) in the AZ cell walls and on the cells distal to the AZ 7 days after induction (Lee et al., 2008). The results suggested the possible involvement of PMEs for the activation of the AZ. However, the study of poinsettia leaf abscission did not examine events after cell separation, and in addition, changes in other cell wall components such as RGI, hemicellulose, and lignin could be more important than pectin (Lee et al., 2008). In tomato, no change in JIM5 or JIM7 was observed during flower or fruit abscission, while increases in xyloglucan, pectic galactan, and arabinan were observed during flower abscission (Iwai et al., 2013). Notably, no changes in signals from any antibodies tested in the previous study were observed during tomato fruit abscission.

In the current study, we provide evidence for a preferential accumulation of JIM5 labeled HG at the edges of separated cells. During development, we observed rough endoplasmic reticulum, mitochondria, and vesicles, in addition to striated cell wall formation at the tips of AZ cells. Together, these results suggest that during development AZ cells undergo polarized oriented cell wall building activity, which culminates with the secretion of HG (JIM5 signal) during abscission in a polarized manner. These functions could either be a part of the mechanism for the abscission process, or part of the defense mechanism to protect the scar after separation. However, these two possible functions are not mutually exclusive and could be linked. Indeed, several lines of evidence suggest an active role for HG in the abscission process. First, the JIM5 epitope increases in the AZ prior to separation and the signal continues to increase after AZ cells separate. Second, pectin accumulation detected in AZ cells prior to separation decreases after separation takes place, suggesting pectin is secreted during abscission. We also observed a high JIM5 signal on both the distal and proximal separated AZ cells, which is not consistent solely with a role for defense, given that a defense response would not be expected in the distal AZ cells of the shed fruit. Finally, a significant decrease in the DME in separated AZ cells closest to the mesocarp suggests that methyl ester groups are being actively removed from HG, possibly by PME activity, in these AZ cell layers, and could explain the high JIM5 signal observed prior to and after AZ cell separation.

During abscission, cell elongation and expansion have been observed but it is unclear whether they are an active part of the abscission process (Wright and Osborne, 1974; Butenko et al., 2003; Patterson and Bleecker, 2004). The current results provide evidence for cell elongation accompanied by a decrease in cell and cell wall width, and an expansion of the middle lamella width in AZ cells that separate. While these changes are significantly quantified in the AZ cells that separate, similar changes do not occur in mesocarp or pedicel cells, again highlighting the differential regulation that controls AZ cell function and fate. Interestingly, during pollen tube and root hair tip growth the polar secretion of HG plays key roles in the elongation process (Rounds and Bezanilla, 2013). The tip growth model proposes that methylated HG is secreted into the wall where it undergoes de-methylesterification by PME, which allows the de-methylesterified HG to form Ca^2+^ crosslinks that results in more rigid cell walls. In the current study, we provide evidence that functional components for tip growth are present in the AZ cells; cell elongation, tip oriented pectin biosynthesis and a decrease in methylesterification, all associated with cells that have undergone separation. While in the tip growth systems cells are elongated, in AZ cells elongation may be a source of HG secretion that plays an active role in cell separation.

## Author contributions

TT and FM devised and participated in all aspects of the study. TT, CJ, and ST coordinated the logistics for study. TT, FM, PR, and CJ performed the ethylene experiments and collected samples for studies. PR, KF, MC, and JV performed the immunolocalization studies. PR, MC, and JV performed the light microscopy and electron microscopy analyses. GM and PR performed the FT-IR microscopy analysis. ST, CJ, PR, and PA participated in the data analysis and critically read the manuscript. TT, JV, FM, and PR participated in writing the article. All authors read and approved the final submitted manuscript.

### Conflict of interest statement

The authors declare that the research was conducted in the absence of any commercial or financial relationships that could be construed as a potential conflict of interest.
