# Supplementary material for: Cellular and Pectin Dynamics during Abscission Zone Development and Ripe Fruit Abscission of the Monocot Oil Palm
Source: Front Plant Sci. 2016 Apr 26;7:540. doi: 10.3389/fpls.2016.00540 (PMC4844998; doi:10.3389/fpls.2016.00540)
Supplement: Supplementary file 1 [file Table1.DOCX]

Supplementary Table 1. Antibodies and stains used in the study.

| **Antibodies and Stains used in Study** | **Epitope or staining character** | **Literature** |
| --- | --- | --- |
| JIM5 | Binds strongly to homogalacturonan the most abundant pectin polymer of galacturonic acid, with a relatively low methylesterfication status and binds weakly to unesterfied oligogalacturonides. | Willats et al., 2000; Clausen et al., 2003 |
| JIM7 | Binds to higher order methylesterified homogalacturonan. | Willats et al., 2000; Clausen et al., 2003 |
| LM7 | Needs at least four unesterified GalA residues between methyl-ester groups to bind. | Clausen et al., 2003 |
| LM8 | Epitope of a xylogalacturonan pectic polysaccharide associated with cell detachment and separation in a wide range of species. | Willats et al., 2004 |
| Ruthenium Red | Selectively binds to the intramolecular spaces of carboxyl groups of pectin. | Sterling, 1970; Hou et al., 1999; Leroux et al., 2007 |
| Alcian Blue | Cationic dye most often used to stain acidic mucosubstances and reported to bind pectin. | Beneš K. 1968. On the Stainability of Plant Cell Walls with Alcian Blue. Biologia Plantarum 10, 334-346. |
| Alcian Blue and periodic acid-Schiff | Combines with characteristics of Alcian Blue with those of periodic acid-Schiff which reacts with polysaccharides such as glycogen, and mucosubstances such as glycoproteins, glycolipids and mucins. | 0 |
| Toluidine Blue | Metachromasia compound that stains lignin and phenols to bluish-green with a pH-independent covalent bonding and non-lignin cell wall components to reddish-violet (acid) (e.g. pectin) and bluish-violet (neutral). | Conrad, 2008 |
| DAPI | Fluorescent nuclear stain that binds to A/T rich DNA repeats. | Kapuscinski J. 1995. DAPI: a DNA-specific fluorescent probe. Biotech Histochem 70, 220-233. |
