# Supplementary material for: Cellular and Pectin Dynamics during Abscission Zone Development and Ripe Fruit Abscission of the Monocot Oil Palm
Source: Front Plant Sci. 2016 Apr 26;7:540. doi: 10.3389/fpls.2016.00540 (PMC4844998; doi:10.3389/fpls.2016.00540)
Supplement: Supplementary file 2 [file Table2.DOCX]

| **Table 2.** The statistical analysis of calculations of cell width in Mesocarp (M), abscission zone (AZ) and pedicel at different developmental stages (DAP 30, 120, 180). | | | | | | | | |
| --- | --- | --- | --- | --- | --- | --- | --- | --- |
| Cell Width | | | | | | | | |
|  | N | Mean | Std. Deviation | Std. Error | 95% Confidence Interval for Mean | | Minimum | Maximum |
|  |  |  |  |  | Lower Bound | Upper Bound |  |  |
| M_30 | 100 | 28.29219 | 4.242297 | .424230 | 27.45043 | 29.13395 | 19.849 | 39.013 |
| P_30 | 100 | 30.94390 | 5.466538 | .546654 | 29.85922 | 32.02858 | 21.260 | 44.045 |
| AZ_30 | 100 | 16.05452 | 3.283155 | .328316 | 15.40307 | 16.70597 | 8.016 | 23.870 |
| M_120 | 100 | 36.66650 | 7.695712 | .769571 | 35.13950 | 38.19350 | 17.720 | 55.946 |
| P_120 | 50 | 32.07796 | 6.342136 | .896913 | 30.27554 | 33.88038 | 19.345 | 46.568 |
| AZ_120 | 100 | 17.84322 | 3.575656 | .357566 | 17.13373 | 18.55271 | 11.180 | 28.570 |
| M_180 | 50 | 34.47572 | 7.074377 | 1.000468 | 32.46520 | 36.48624 | 22.783 | 50.678 |
| P_180 | 50 | 34.18784 | 6.278853 | .887964 | 32.40341 | 35.97227 | 21.260 | 47.539 |
| AZ_180 | 50 | 21.87328 | 4.452208 | .629637 | 20.60798 | 23.13858 | 13.643 | 33.273 |
| Total | 700 | 27.30110 | 9.234782 | .349042 | 26.61581 | 27.98640 | 8.016 | 55.946 |

**Table 3.** The test of homogeneity of variances of cell width of tissues and developmental stages. The Levene test rejects the hypothesis that the variances are equal.

| Cell Width | | | |
| --- | --- | --- | --- |
| Levene Statistic | df1 | df2 | Sig. |
| 13.409 | 8 | 691 | .000 |

**Table 4.** The ANOVA test of cell width of tissues and developmental stages. The result rejects the hypothesis that there is no difference between the groups.

| Cell Width | | | | | |
| --- | --- | --- | --- | --- | --- |
|  | Sum of Squares | df | Mean Square | F | Sig. |
| Between Groups | 39349.101 | 8 | 4918.638 | 167.738 | .000 |
| Within Groups | 20262.453 | 691 | 29.323 |  |  |
| Total | 59611.554 | 699 |  |  |  |

| **Table 5.** (Cont.) The Post Hoc tests of cell width of tissues and developmental stages by using DunnettT3. | | | | | | |
| --- | --- | --- | --- | --- | --- | --- |
| Cell Width  Dunnett T3 | | | | | | |
| (I) Tissue_DAP | (J) Tissue_DAP | Mean Difference (I-J) | Std. Error | Sig. | 95% Confidence Interval | |
|  |  |  |  |  | Lower Bound | Upper Bound |
| M_30 | P_30 | -2.651710^*^ | .691955 | .006 | -4.89053 | -.41289 |
|  | AZ_30 | 12.237670^*^ | .536434 | .000 | 10.50201 | 13.97333 |
|  | M_120 | -8.374310^*^ | .878755 | .000 | -11.22603 | -5.52259 |
|  | P_120 | -3.785770^*^ | .992182 | .010 | -7.06984 | -.50170 |
|  | AZ_120 | 10.448970^*^ | .554819 | .000 | 8.65465 | 12.24329 |
|  | M_180 | -6.183530^*^ | 1.086696 | .000 | -9.78927 | -2.57779 |
|  | P_180 | -5.895650^*^ | .984099 | .000 | -9.15220 | -2.63910 |
|  | AZ_180 | 6.418910^*^ | .759219 | .000 | 3.92791 | 8.90991 |
| P_30 | M_30 | 2.651710^*^ | .691955 | .006 | .41289 | 4.89053 |
|  | AZ_30 | 14.889380^*^ | .637669 | .000 | 12.82181 | 16.95695 |
|  | M_120 | -5.722600^*^ | .943965 | .000 | -8.77871 | -2.66649 |
|  | P_120 | -1.134060 | 1.050373 | 1.000 | -4.58902 | 2.32090 |
|  | AZ_120 | 13.100680^*^ | .653210 | .000 | 10.98442 | 15.21694 |
|  | M_180 | -3.531820 | 1.140073 | .090 | -7.29220 | .22856 |
|  | P_180 | -3.243940 | 1.042742 | .085 | -6.67293 | .18505 |
|  | AZ_180 | 9.070620^*^ | .833831 | .000 | 6.35024 | 11.79100 |
| AZ_30 | M_30 | -12.237670^*^ | .536434 | .000 | -13.97333 | -10.50201 |
|  | P_30 | -14.889380^*^ | .637669 | .000 | -16.95695 | -12.82181 |
|  | M_120 | -20.611980^*^ | .836679 | .000 | -23.33421 | -17.88975 |
|  | P_120 | -16.023440^*^ | .955115 | .000 | -19.20190 | -12.84498 |
|  | AZ_120 | -1.788700^*^ | .485432 | .011 | -3.35817 | -.21923 |
|  | M_180 | -18.421200^*^ | 1.052961 | .000 | -21.93189 | -14.91051 |
|  | P_180 | -18.133320^*^ | .946716 | .000 | -21.28324 | -14.98340 |
|  | AZ_180 | -5.818760^*^ | .710094 | .000 | -8.16363 | -3.47389 |
| M_120 | M_30 | 8.374310^*^ | .878755 | .000 | 5.52259 | 11.22603 |
|  | P_30 | 5.722600^*^ | .943965 | .000 | 2.66649 | 8.77871 |
|  | AZ_30 | 20.611980^*^ | .836679 | .000 | 17.88975 | 23.33421 |
|  | P_120 | 4.588540^*^ | 1.181818 | .006 | .73198 | 8.44510 |
|  | AZ_120 | 18.823280^*^ | .848583 | .000 | 16.06465 | 21.58191 |
|  | M_180 | 2.190780 | 1.262211 | .946 | -1.93756 | 6.31912 |
|  | P_180 | 2.478660 | 1.175040 | .717 | -1.35505 | 6.31237 |
|  | AZ_180 | 14.793220^*^ | .994325 | .000 | 11.56290 | 18.02354 |
| P120 | M_30 | 3.785770^*^ | .992182 | .010 | .50170 | 7.06984 |
|  | P_30 | 1.134060 | 1.050373 | 1.000 | -2.32090 | 4.58902 |
|  | AZ_30 | 16.023440^*^ | .955115 | .000 | 12.84498 | 19.20190 |
|  | M_120 | -4.588540^*^ | 1.181818 | .006 | -8.44510 | -.73198 |
|  | AZ_120 | 14.234740^*^ | .965560 | .000 | 11.02682 | 17.44266 |
|  | M_180 | -2.397760 | 1.343648 | .927 | -6.80262 | 2.00710 |
|  | P_180 | -2.109880 | 1.262115 | .964 | -6.24614 | 2.02638 |
|  | AZ_180 | 10.204680^*^ | 1.095854 | .000 | 6.60211 | 13.80725 |
| AZ_120 | M_30 | -10.448970^*^ | .554819 | .000 | -12.24329 | -8.65465 |
|  | P_30 | -13.100680^*^ | .653210 | .000 | -15.21694 | -10.98442 |
|  | AZ_30 | 1.788700^*^ | .485432 | .011 | .21923 | 3.35817 |
|  | M_120 | -18.823280^*^ | .848583 | .000 | -21.58191 | -16.06465 |
|  | P_120 | -14.234740^*^ | .965560 | .000 | -17.44266 | -11.02682 |
|  | M_180 | -16.632500^*^ | 1.062445 | .000 | -20.16967 | -13.09533 |
|  | P_180 | -16.344620^*^ | .957253 | .000 | -19.52429 | -13.16495 |
|  | AZ_180 | -4.030060^*^ | .724083 | .000 | -6.41603 | -1.64409 |
| M_180 | M_30 | 6.183530^*^ | 1.086696 | .000 | 2.57779 | 9.78927 |
|  | P_30 | 3.531820 | 1.140073 | .090 | -.22856 | 7.29220 |
|  | AZ_30 | 18.421200^*^ | 1.052961 | .000 | 14.91051 | 21.93189 |
|  | M_120 | -2.190780 | 1.262211 | .946 | -6.31912 | 1.93756 |
|  | P_120 | 2.397760 | 1.343648 | .927 | -2.00710 | 6.80262 |
|  | AZ_120 | 16.632500^*^ | 1.062445 | .000 | 13.09533 | 20.16967 |
|  | P_180 | .287880 | 1.337691 | 1.000 | -4.09772 | 4.67348 |
|  | AZ_180 | 12.602440^*^ | 1.182108 | .000 | 8.70880 | 16.49608 |
| P_180 | M_30 | 5.895650^*^ | .984099 | .000 | 2.63910 | 9.15220 |
|  | P_30 | 3.243940 | 1.042742 | .085 | -.18505 | 6.67293 |
|  | AZ_30 | 18.133320^*^ | .946716 | .000 | 14.98340 | 21.28324 |
|  | M_120 | -2.478660 | 1.175040 | .717 | -6.31237 | 1.35505 |
|  | P_120 | 2.109880 | 1.262115 | .964 | -2.02638 | 6.24614 |
|  | AZ_120 | 16.344620^*^ | .957253 | .000 | 13.16495 | 19.52429 |
|  | M_180 | -.287880 | 1.337691 | 1.000 | -4.67348 | 4.09772 |
|  | AZ_180 | 12.314560^*^ | 1.088542 | .000 | 8.73660 | 15.89252 |
| AZ_180 | M_30 | -6.418910^*^ | .759219 | .000 | -8.90991 | -3.92791 |
|  | P_30 | -9.070620^*^ | .833831 | .000 | -11.79100 | -6.35024 |
|  | AZ_30 | 5.818760^*^ | .710094 | .000 | 3.47389 | 8.16363 |
|  | M_120 | -14.793220^*^ | .994325 | .000 | -18.02354 | -11.56290 |
|  | P_120 | -10.204680^*^ | 1.095854 | .000 | -13.80725 | -6.60211 |
|  | AZ_120 | 4.030060^*^ | .724083 | .000 | 1.64409 | 6.41603 |
|  | M_180 | -12.602440^*^ | 1.182108 | .000 | -16.49608 | -8.70880 |
|  | P_180 | -12.314560^*^ | 1.088542 | .000 | -15.89252 | -8.73660 |
| *. The mean difference is significant at the 0.05 level. | | | | | | |

**Table 6.** The statistical descriptives of cell wall width of tissues and developmental stages.

| Cell Wall Width | | | | | | | | |
| --- | --- | --- | --- | --- | --- | --- | --- | --- |
|  | N | Mean | Std. Deviation | Std. Error | 95% Confidence Interval for Mean | | Minimum | Maximum |
|  |  |  |  |  | Lower Bound | Upper Bound |  |  |
| M_30 | 30 | .59067 | .185528 | .033873 | .52139 | .65994 | .303 | 1.282 |
| P_30 | 20 | 1.04935 | .260594 | .058271 | .92739 | 1.17131 | .647 | 1.538 |
| AZ_30 | 27 | .90837 | .170138 | .032743 | .84107 | .97567 | .555 | 1.182 |
| M_120 | 50 | 1.25174 | .276608 | .039118 | 1.17313 | 1.33035 | .848 | 2.287 |
| P_120 | 40 | 1.25580 | .286758 | .045340 | 1.16409 | 1.34751 | .795 | 2.277 |
| AZ_120 | 31 | 2.12432 | .502505 | .090253 | 1.94000 | 2.30864 | 1.414 | 3.704 |
| M_180 | 25 | 2.59396 | .433139 | .086628 | 2.41517 | 2.77275 | 1.656 | 3.794 |
| P_180 | 25 | 2.60868 | .332913 | .066583 | 2.47126 | 2.74610 | 2.048 | 3.321 |
| AZ_180 | 25 | 2.58044 | .392993 | .078599 | 2.41822 | 2.74266 | 2.051 | 3.349 |
| Total | 273 | 1.59884 | .793993 | .048055 | 1.50423 | 1.69345 | .303 | 3.794 |

**Table 7.** The test of homogeneity of variances of cell wall width of tissues and developmental stages. The Levene test rejects the hypothesis that the variances are equal.

| Cell Wall Width | | | |
| --- | --- | --- | --- |
| Levene Statistic | df1 | df2 | Sig. |
| 5.045 | 8 | 264 | .000 |

**Table 8.** The ANOVA test of cell wall width of tissues and developmental stages. The result rejects the hypothesis that there is no difference between the groups.

| Cell Wall Width | | | | | |
| --- | --- | --- | --- | --- | --- |
|  | Sum of Squares | df | Mean Square | F | Sig. |
| Between Groups | 143.034 | 8 | 17.879 | 165.958 | .000 |
| Within Groups | 28.442 | 264 | .108 |  |  |
| Total | 171.476 | 272 |  |  |  |

| **Table 9.** (Cont.) The Post Hoc tests of cell wall width of tissues and developmental stages by using DunnettT3. | | | | | | |
| --- | --- | --- | --- | --- | --- | --- |
| Cell Wall Width  Dunnett T3 | | | | | | |
| (I) Tissue_DAP | (J) Tissue_DAP | Mean Difference (I-J) | Std. Error | Sig. | 95% Confidence Interval | |
|  |  |  |  |  | Lower Bound | Upper Bound |
| M_30 | P_30 | -.458683^*^ | .067400 | .000 | -.69231 | -.22506 |
|  | AZ_30 | -.317704^*^ | .047111 | .000 | -.47538 | -.16003 |
|  | M_120 | -.661073^*^ | .051745 | .000 | -.83191 | -.49024 |
|  | P_120 | -.665133^*^ | .056596 | .000 | -.85297 | -.47730 |
|  | AZ_120 | -1.533656^*^ | .096400 | .000 | -1.86308 | -1.20423 |
|  | M_180 | -2.003293^*^ | .093015 | .000 | -2.32600 | -1.68059 |
|  | P_180 | -2.018013^*^ | .074703 | .000 | -2.27437 | -1.76165 |
|  | AZ_180 | -1.989773^*^ | .085587 | .000 | -2.28557 | -1.69397 |
| P_30 | M_30 | .458683^*^ | .067400 | .000 | .22506 | .69231 |
|  | AZ_30 | .140980 | .066840 | .709 | -.09131 | .37327 |
|  | M_120 | -.202390 | .070183 | .189 | -.44275 | .03797 |
|  | P_120 | -.206450 | .073832 | .223 | -.45740 | .04450 |
|  | AZ_120 | -1.074973^*^ | .107429 | .000 | -1.43734 | -.71260 |
|  | M_180 | -1.544610^*^ | .104402 | .000 | -1.90019 | -1.18903 |
|  | P_180 | -1.559330^*^ | .088480 | .000 | -1.85943 | -1.25923 |
|  | AZ_180 | -1.531090^*^ | .097843 | .000 | -1.86356 | -1.19862 |
| AZ_30 | M_30 | .317704^*^ | .047111 | .000 | .16003 | .47538 |
|  | P_30 | -.140980 | .066840 | .709 | -.37327 | .09131 |
|  | M_120 | -.343370^*^ | .051013 | .000 | -.51205 | -.17469 |
|  | P_120 | -.347430^*^ | .055927 | .000 | -.53334 | -.16152 |
|  | AZ_120 | -1.215952^*^ | .096009 | .000 | -1.54440 | -.88751 |
|  | M_180 | -1.685590^*^ | .092609 | .000 | -2.00734 | -1.36384 |
|  | P_180 | -1.700310^*^ | .074198 | .000 | -1.95542 | -1.44520 |
|  | AZ_180 | -1.672070^*^ | .085146 | .000 | -1.96682 | -1.37732 |
| M_120 | M_30 | .661073^*^ | .051745 | .000 | .49024 | .83191 |
|  | P_30 | .202390 | .070183 | .189 | -.03797 | .44275 |
|  | AZ_30 | .343370^*^ | .051013 | .000 | .17469 | .51205 |
|  | P_120 | -.004060 | .059883 | 1.000 | -.20132 | .19320 |
|  | AZ_120 | -.872583^*^ | .098365 | .000 | -1.20697 | -.53819 |
|  | M_180 | -1.342220^*^ | .095051 | .000 | -1.66975 | -1.01469 |
|  | P_180 | -1.356940^*^ | .077224 | .000 | -1.61962 | -1.09426 |
|  | AZ_180 | -1.328700^*^ | .087795 | .000 | -1.62984 | -1.02756 |
| P_120 | M_30 | .665133^*^ | .056596 | .000 | .47730 | .85297 |
|  | P_30 | .206450 | .073832 | .223 | -.04450 | .45740 |
|  | AZ_30 | .347430^*^ | .055927 | .000 | .16152 | .53334 |
|  | M_120 | .004060 | .059883 | 1.000 | -.19320 | .20132 |
|  | AZ_120 | -.868523^*^ | .101001 | .000 | -1.21023 | -.52682 |
|  | M_180 | -1.338160^*^ | .097776 | .000 | -1.67292 | -1.00340 |
|  | P_180 | -1.352880^*^ | .080554 | .000 | -1.62521 | -1.08055 |
|  | AZ_180 | -1.324640^*^ | .090739 | .000 | -1.63385 | -1.01543 |
| AZ_120 | M_30 | 1.533656^*^ | .096400 | .000 | 1.20423 | 1.86308 |
|  | P_30 | 1.074973^*^ | .107429 | .000 | .71260 | 1.43734 |
|  | AZ_30 | 1.215952^*^ | .096009 | .000 | .88751 | 1.54440 |
|  | M_120 | .872583^*^ | .098365 | .000 | .53819 | 1.20697 |
|  | P_120 | .868523^*^ | .101001 | .000 | .52682 | 1.21023 |
|  | M_180 | -.469637^*^ | .125100 | .015 | -.88880 | -.05047 |
|  | P_180 | -.484357^*^ | .112155 | .003 | -.86069 | -.10803 |
|  | AZ_180 | -.456117^*^ | .119680 | .013 | -.85704 | -.05520 |
| M_180 | M_30 | 2.003293^*^ | .093015 | .000 | 1.68059 | 2.32600 |
|  | P_30 | 1.544610^*^ | .104402 | .000 | 1.18903 | 1.90019 |
|  | AZ_30 | 1.685590^*^ | .092609 | .000 | 1.36384 | 2.00734 |
|  | M_120 | 1.342220^*^ | .095051 | .000 | 1.01469 | 1.66975 |
|  | P_120 | 1.338160^*^ | .097776 | .000 | 1.00340 | 1.67292 |
|  | AZ_120 | .469637^*^ | .125100 | .015 | .05047 | .88880 |
|  | P_180 | -.014720 | .109259 | 1.000 | -.38428 | .35484 |
|  | AZ_180 | .013520 | .116971 | 1.000 | -.38090 | .40794 |
| P_180 | M_30 | 2.018013^*^ | .074703 | .000 | 1.76165 | 2.27437 |
|  | P_30 | 1.559330^*^ | .088480 | .000 | 1.25923 | 1.85943 |
|  | AZ_30 | 1.700310^*^ | .074198 | .000 | 1.44520 | 1.95542 |
|  | M_120 | 1.356940^*^ | .077224 | .000 | 1.09426 | 1.61962 |
|  | P_120 | 1.352880^*^ | .080554 | .000 | 1.08055 | 1.62521 |
|  | AZ_120 | .484357^*^ | .112155 | .003 | .10803 | .86069 |
|  | M_180 | .014720 | .109259 | 1.000 | -.35484 | .38428 |
|  | AZ_180 | .028240 | .103010 | 1.000 | -.31944 | .37592 |
| AZ_180 | M_30 | 1.989773^*^ | .085587 | .000 | 1.69397 | 2.28557 |
|  | P_30 | 1.531090^*^ | .097843 | .000 | 1.19862 | 1.86356 |
|  | AZ_30 | 1.672070^*^ | .085146 | .000 | 1.37732 | 1.96682 |
|  | M_120 | 1.328700^*^ | .087795 | .000 | 1.02756 | 1.62984 |
|  | P_120 | 1.324640^*^ | .090739 | .000 | 1.01543 | 1.63385 |
|  | AZ_120 | .456117^*^ | .119680 | .013 | .05520 | .85704 |
|  | M_180 | -.013520 | .116971 | 1.000 | -.40794 | .38090 |
|  | P_180 | -.028240 | .103010 | 1.000 | -.37592 | .31944 |
| *. The mean difference is significant at the 0.05 level. | | | | | | |

**Table 10.** The statistical descriptives of middle lamella width of tissues and developmental stages.

| Middle Lamella Width | | | | | | | | |
| --- | --- | --- | --- | --- | --- | --- | --- | --- |
|  | N | Mean | Std. Deviation | Std. Error | 95% Confidence Interval for Mean | | Minimum | Maximum |
|  |  |  |  |  | Lower Bound | Upper Bound |  |  |
| M_120 | 30 | .57737 | .137432 | .025092 | .52605 | .62868 | .328 | 1.006 |
| P_120 | 18 | .65067 | .197370 | .046521 | .55252 | .74882 | .335 | 1.082 |
| AZ_120 | 25 | .59464 | .212990 | .042598 | .50672 | .68256 | .319 | 1.178 |
| M_180 | 20 | .92535 | .257135 | .057497 | .80501 | 1.04569 | .357 | 1.511 |
| P_180 | 20 | .99270 | .378224 | .084574 | .81569 | 1.16971 | .398 | 1.713 |
| AZ_180 | 25 | .94480 | .253818 | .050764 | .84003 | 1.04957 | .543 | 1.500 |
| Total | 138 | .76725 | .298352 | .025397 | .71702 | .81747 | .319 | 1.713 |

**Table 11.** The test of homogeneity of variances of middle lamella width of tissues and developmental stage. The Levene test rejects the hypothesis that the variances are equal.

| Middle Lamella Width | | | |
| --- | --- | --- | --- |
| Levene Statistic | df1 | df2 | Sig. |
| 5.154 | 5 | 132 | .000 |

**Table 12.** The ANOVA test of middle lamella width of tissues and developmental stages. The result rejects the hypothesis that there is no difference between the groups.

| Middle Lamella Width | | | | | |
| --- | --- | --- | --- | --- | --- |
|  | Sum of Squares | df | Mean Square | F | Sig. |
| Between Groups | 4.376 | 5 | .875 | 14.774 | .000 |
| Within Groups | 7.819 | 132 | .059 |  |  |
| Total | 12.195 | 137 |  |  |  |

| **Table 13.** (Cont.) The Post Hoc tests of middle lamella of tissues and developmental stages by using DunnettT3. | | | | | | |
| --- | --- | --- | --- | --- | --- | --- |
| Middle Lamella Width  Dunnett T3 | | | | | | |
| (I) Tissue_DAP | (J) Tissue_DAP | Mean Difference (I-J) | Std. Error | Sig. | 95% Confidence Interval | |
|  |  |  |  |  | Lower Bound | Upper Bound |
| M_120 | P_120 | -.073300 | .052856 | .917 | -.24172 | .09512 |
|  | AZ_120 | -.017273 | .049439 | 1.000 | -.17062 | .13607 |
|  | M_180 | -.347983^*^ | .062734 | .000 | -.54832 | -.14764 |
|  | P_180 | -.415333^*^ | .088217 | .002 | -.70137 | -.12929 |
|  | AZ_180 | -.367433^*^ | .056626 | .000 | -.54427 | -.19059 |
| P_120 | M_120 | .073300 | .052856 | .917 | -.09512 | .24172 |
|  | AZ_120 | .056027 | .063077 | .998 | -.13999 | .25204 |
|  | M_180 | -.274683^*^ | .073960 | .010 | -.50576 | -.04361 |
|  | P_180 | -.342033^*^ | .096524 | .019 | -.64760 | -.03646 |
|  | AZ_180 | -.294133^*^ | .068856 | .002 | -.50737 | -.08090 |
| AZ_120 | M_120 | .017273 | .049439 | 1.000 | -.13607 | .17062 |
|  | P_120 | -.056027 | .063077 | .998 | -.25204 | .13999 |
|  | M_180 | -.330710^*^ | .071558 | .001 | -.55364 | -.10778 |
|  | P_180 | -.398060^*^ | .094696 | .003 | -.69853 | -.09759 |
|  | AZ_180 | -.350160^*^ | .066269 | .000 | -.55393 | -.14639 |
| M_180 | M_120 | .347983^*^ | .062734 | .000 | .14764 | .54832 |
|  | P_120 | .274683^*^ | .073960 | .010 | .04361 | .50576 |
|  | AZ_120 | .330710^*^ | .071558 | .001 | .10778 | .55364 |
|  | P_180 | -.067350 | .102267 | 1.000 | -.38792 | .25322 |
|  | AZ_180 | -.019450 | .076700 | 1.000 | -.25700 | .21810 |
| P_180 | M_120 | .415333^*^ | .088217 | .002 | .12929 | .70137 |
|  | P_120 | .342033^*^ | .096524 | .019 | .03646 | .64760 |
|  | AZ_120 | .398060^*^ | .094696 | .003 | .09759 | .69853 |
|  | M_180 | .067350 | .102267 | 1.000 | -.25322 | .38792 |
|  | AZ_180 | .047900 | .098639 | 1.000 | -.26235 | .35815 |
| AZ_180 | M_120 | .367433^*^ | .056626 | .000 | .19059 | .54427 |
|  | P_120 | .294133^*^ | .068856 | .002 | .08090 | .50737 |
|  | AZ_120 | .350160^*^ | .066269 | .000 | .14639 | .55393 |
|  | M_180 | .019450 | .076700 | 1.000 | -.21810 | .25700 |
|  | P_180 | -.047900 | .098639 | 1.000 | -.35815 | .26235 |
| *. The mean difference is significant at the 0.05 level. | | | | | | |
