# Supplementary material for: Cellular and Pectin Dynamics during Abscission Zone Development and Ripe Fruit Abscission of the Monocot Oil Palm
Source: Front Plant Sci. 2016 Apr 26;7:540. doi: 10.3389/fpls.2016.00540 (PMC4844998; doi:10.3389/fpls.2016.00540)
Supplement: Supplementary file 3 [file Table3.DOCX]

**Table 14.** The statistical descriptives of cell width of tissues before and after separations.

| Cell Width before and after separation | | | | | | | | |
| --- | --- | --- | --- | --- | --- | --- | --- | --- |
|  | N | Mean | Std. Deviation | Std. Error | 95% Confidence Interval for Mean | | Minimum | Maximum |
|  |  |  |  |  | Lower Bound | Upper Bound |  |  |
| M_180 | 50 | 34.47572 | 7.074377 | 1.000468 | 32.46520 | 36.48624 | 22.783 | 50.678 |
| P_180 | 50 | 34.18784 | 6.278853 | .887964 | 32.40341 | 35.97227 | 21.260 | 47.539 |
| AZ_180 | 50 | 21.87328 | 4.452208 | .629637 | 20.60798 | 23.13858 | 13.643 | 33.273 |
| M_180+ | 50 | 35.53978 | 7.759846 | 1.097408 | 33.33446 | 37.74510 | 17.000 | 53.502 |
| P_180+ | 50 | 34.12092 | 7.263392 | 1.027199 | 32.05669 | 36.18515 | 22.472 | 53.000 |
| AZsep_180+ | 30 | 19.42217 | 3.576062 | .652897 | 18.08684 | 20.75749 | 13.200 | 26.130 |
| AZunsep_180+ | 30 | 17.99003 | 3.984025 | .727380 | 16.50237 | 19.47769 | 13.306 | 31.116 |
| Total | 310 | 29.45885 | 9.326772 | .529725 | 28.41652 | 30.50117 | 13.200 | 53.502 |

**Table 15.** The test of homogeneity of variances of cell width of tissues before and after separation. The Levene test rejects the hypothesis that the variances are equal.

| Cell Width before and after separation | | | |
| --- | --- | --- | --- |
| Levene Statistic | df1 | df2 | Sig. |
| 6.091 | 6 | 303 | .000 |

**Table 16.** The ANOVA test of cell width of tissues and developmental stages. The result rejects the hypothesis that there is no difference between the groups.

| Cell Width before and after separation | | | | | |
| --- | --- | --- | --- | --- | --- |
|  | Sum of Squares | df | Mean Square | F | Sig. |
| Between Groups | 15157.354 | 6 | 2526.226 | 65.299 | .000 |
| Within Groups | 11722.146 | 303 | 38.687 |  |  |
| Total | 26879.500 | 309 |  |  |  |

| **Table 17.** (Cont.) The Post Hoc tests of cell width of tissues before and after separation by using DunnettT3. | | | | | | |
| --- | --- | --- | --- | --- | --- | --- |
| Cell width before and after separation  Dunnett T3 | | | | | | |
| (I) Tissue_DAP | (J) Tissue_DAP | Mean Difference (I-J) | Std. Error | Sig. | 95% Confidence Interval | |
|  |  |  |  |  | Lower Bound | Upper Bound |
| M_180 | P_180 | .287880 | 1.337691 | 1.000 | -3.86929 | 4.44505 |
|  | AZ_180 | 12.602440^*^ | 1.182108 | .000 | 8.91303 | 16.29185 |
|  | M_180+ | -1.064060 | 1.485005 | 1.000 | -5.67840 | 3.55028 |
|  | P_180+ | .354800 | 1.433901 | 1.000 | -4.09989 | 4.80949 |
|  | AZsep_180+ | 15.053553^*^ | 1.194659 | .000 | 11.31592 | 18.79118 |
|  | AZunsep_180+ | 16.485687^*^ | 1.236939 | .000 | 12.61818 | 20.35319 |
| P_180 | M_180 | -.287880 | 1.337691 | 1.000 | -4.44505 | 3.86929 |
|  | AZ_180 | 12.314560^*^ | 1.088542 | .000 | 8.92369 | 15.70543 |
|  | M_180+ | -1.351940 | 1.411660 | 1.000 | -5.74218 | 3.03830 |
|  | P_180+ | .066920 | 1.357799 | 1.000 | -4.15345 | 4.28729 |
|  | AZsep_180+ | 14.765673^*^ | 1.102159 | .000 | 11.31969 | 18.21165 |
|  | AZunsep_180+ | 16.197807^*^ | 1.147851 | .000 | 12.60875 | 19.78686 |
| AZ_180 | M_180 | -12.602440^*^ | 1.182108 | .000 | -16.29185 | -8.91303 |
|  | P_180 | -12.314560^*^ | 1.088542 | .000 | -15.70543 | -8.92369 |
|  | M_180+ | -13.666500^*^ | 1.265206 | .000 | -17.62187 | -9.71113 |
|  | P_180+ | -12.247640^*^ | 1.204815 | .000 | -16.00967 | -8.48561 |
|  | AZsep_180+ | 2.451113 | .907037 | .160 | -.39273 | 5.29495 |
|  | AZunsep_180+ | 3.883247^*^ | .962042 | .003 | .85951 | 6.90698 |
| M_180+ | M_180 | 1.064060 | 1.485005 | 1.000 | -3.55028 | 5.67840 |
|  | P_180 | 1.351940 | 1.411660 | 1.000 | -3.03830 | 5.74218 |
|  | AZ_180 | 13.666500^*^ | 1.265206 | .000 | 9.71113 | 17.62187 |
|  | P_180+ | 1.418860 | 1.503144 | 1.000 | -3.25137 | 6.08909 |
|  | AZsep_180+ | 16.117613^*^ | 1.276941 | .000 | 12.11892 | 20.11630 |
|  | AZunsep_180+ | 17.549747^*^ | 1.316581 | .000 | 13.43117 | 21.66832 |
| P_180+ | M_180 | -.354800 | 1.433901 | 1.000 | -4.80949 | 4.09989 |
|  | P_180 | -.066920 | 1.357799 | 1.000 | -4.28729 | 4.15345 |
|  | AZ_180 | 12.247640^*^ | 1.204815 | .000 | 8.48561 | 16.00967 |
|  | M_180+ | -1.418860 | 1.503144 | 1.000 | -6.08909 | 3.25137 |
|  | AZsep_180+ | 14.698753^*^ | 1.217132 | .000 | 10.88994 | 18.50756 |
|  | AZunsep_180+ | 16.130887^*^ | 1.258658 | .000 | 12.19508 | 20.06669 |
| AZsep_180+ | M_180 | -15.053553^*^ | 1.194659 | .000 | -18.79118 | -11.31592 |
|  | P_180 | -14.765673^*^ | 1.102159 | .000 | -18.21165 | -11.31969 |
|  | AZ_180 | -2.451113 | .907037 | .160 | -5.29495 | .39273 |
|  | M_180+ | -16.117613^*^ | 1.276941 | .000 | -20.11630 | -12.11892 |
|  | P_180+ | -14.698753^*^ | 1.217132 | .000 | -18.50756 | -10.88994 |
|  | AZunsep_180+ | 1.432133 | .977423 | .951 | -1.65805 | 4.52231 |
| AZunsep_180+ | M_180 | -16.485687^*^ | 1.236939 | .000 | -20.35319 | -12.61818 |
|  | P_180 | -16.197807^*^ | 1.147851 | .000 | -19.78686 | -12.60875 |
|  | AZ_180 | -3.883247^*^ | .962042 | .003 | -6.90698 | -.85951 |
|  | M_180+ | -17.549747^*^ | 1.316581 | .000 | -21.66832 | -13.43117 |
|  | P_180+ | -16.130887^*^ | 1.258658 | .000 | -20.06669 | -12.19508 |
|  | AZsep_180+ | -1.432133 | .977423 | .951 | -4.52231 | 1.65805 |
| *. The mean difference is significant at the 0.05 level. | | | | | | |

**Table 18.** The statistical descriptives of cell wall width of tissues before and after separation.

| Cell wall width before and after separation | | | | | | | | |
| --- | --- | --- | --- | --- | --- | --- | --- | --- |
|  | N | Mean | Std. Deviation | Std. Error | 95% Confidence Interval for Mean | | Minimum | Maximum |
|  |  |  |  |  | Lower Bound | Upper Bound |  |  |
| M_180 | 25 | 2.59396 | .433139 | .086628 | 2.41517 | 2.77275 | 1.656 | 3.794 |
| P_180 | 25 | 2.60868 | .332913 | .066583 | 2.47126 | 2.74610 | 2.048 | 3.321 |
| AZ_180 | 25 | 2.58044 | .392993 | .078599 | 2.41822 | 2.74266 | 2.051 | 3.349 |
| M_180+ | 25 | 2.79788 | .660131 | .132026 | 2.52539 | 3.07037 | 1.638 | 3.980 |
| P_180+ | 35 | 2.45926 | .558366 | .094381 | 2.26745 | 2.65106 | 1.423 | 3.393 |
| AZsep_180+ | 63 | 1.75813 | .447923 | .056433 | 1.64532 | 1.87093 | 1.067 | 3.185 |
| AZunsep_180+ | 43 | 1.83953 | .410827 | .062651 | 1.71310 | 1.96597 | .907 | 2.986 |
| Total | 241 | 2.24257 | .618703 | .039854 | 2.16406 | 2.32108 | .907 | 3.980 |

**Table 19.** The test of homogeneity of variances of cell wall width of tissues before and after separation. The Levene test rejects the hypothesis that the variances are equal.

| Cell wall width before and after separation | | | |
| --- | --- | --- | --- |
| Levene Statistic | df1 | df2 | Sig. |
| 2.834 | 6 | 234 | .011 |

**Table 20.** The ANOVA test of cell wall width of tissues before and after separation. The result rejects the hypothesis that there is no difference between the groups.

| Cell wall width before and after separation | | | | | |
| --- | --- | --- | --- | --- | --- |
|  | Sum of Squares | df | Mean Square | F | Sig. |
| Between Groups | 40.414 | 6 | 6.736 | 30.631 | .000 |
| Within Groups | 51.456 | 234 | .220 |  |  |
| Total | 91.870 | 240 |  |  |  |

| **Table 21.** (Cont.) The Post Hoc tests of cell wall width of tissues before and after separation by using DunnettT3. | | | | | | |
| --- | --- | --- | --- | --- | --- | --- |
| Cell wall width before and after separation  Dunnett T3 | | | | | | |
| (I) Tissue_DAP | (J) Tissue_DAP | Mean Difference (I-J) | Std. Error | Sig. | 95% Confidence Interval | |
|  |  |  |  |  | Lower Bound | Upper Bound |
| M_180 | P_180 | -.014720 | .109259 | 1.000 | -.36416 | .33472 |
|  | AZ_180 | .013520 | .116971 | 1.000 | -.35951 | .38655 |
|  | M_180+ | -.203920 | .157909 | .984 | -.71130 | .30346 |
|  | P_180+ | .134703 | .128110 | .999 | -.27026 | .53967 |
|  | AZsep_180+ | .835833^*^ | .103388 | .000 | .50537 | 1.16630 |
|  | AZunsep_180+ | .754425^*^ | .106909 | .000 | .41369 | 1.09516 |
| P_180 | M_180 | .014720 | .109259 | 1.000 | -.33472 | .36416 |
|  | AZ_180 | .028240 | .103010 | 1.000 | -.30056 | .35704 |
|  | M_180+ | -.189200 | .147865 | .984 | -.66899 | .29059 |
|  | P_180+ | .149423 | .115503 | .985 | -.21599 | .51483 |
|  | AZsep_180+ | .850553^*^ | .087281 | .000 | .57495 | 1.12616 |
|  | AZunsep_180+ | .769145^*^ | .091424 | .000 | .48043 | 1.05786 |
| AZ_180 | M_180 | -.013520 | .116971 | 1.000 | -.38655 | .35951 |
|  | P_180 | -.028240 | .103010 | 1.000 | -.35704 | .30056 |
|  | M_180+ | -.217440 | .153651 | .961 | -.71285 | .27797 |
|  | P_180+ | .121183 | .122823 | .999 | -.26694 | .50931 |
|  | AZsep_180+ | .822313^*^ | .096760 | .000 | .51450 | 1.13013 |
|  | AZunsep_180+ | .740905^*^ | .100513 | .000 | .42179 | 1.06002 |
| M_180+ | M_180 | .203920 | .157909 | .984 | -.30346 | .71130 |
|  | P_180 | .189200 | .147865 | .984 | -.29059 | .66899 |
|  | AZ_180 | .217440 | .153651 | .961 | -.27797 | .71285 |
|  | P_180+ | .338623 | .162292 | .558 | -.17968 | .85692 |
|  | AZsep_180+ | 1.039753^*^ | .143581 | .000 | .57164 | 1.50786 |
|  | AZunsep_180+ | .958345^*^ | .146137 | .000 | .48375 | 1.43294 |
| P_180+ | M_180 | -.134703 | .128110 | .999 | -.53967 | .27026 |
|  | P_180 | -.149423 | .115503 | .985 | -.51483 | .21599 |
|  | AZ_180 | -.121183 | .122823 | .999 | -.50931 | .26694 |
|  | M_180+ | -.338623 | .162292 | .558 | -.85692 | .17968 |
|  | AZsep_180+ | .701130^*^ | .109966 | .000 | .35377 | 1.04849 |
|  | AZunsep_180+ | .619722^*^ | .113282 | .000 | .26248 | .97697 |
| AZsep_180+ | M_180 | -.835833^*^ | .103388 | .000 | -1.16630 | -.50537 |
|  | P_180 | -.850553^*^ | .087281 | .000 | -1.12616 | -.57495 |
|  | AZ_180 | -.822313^*^ | .096760 | .000 | -1.13013 | -.51450 |
|  | M_180+ | -1.039753^*^ | .143581 | .000 | -1.50786 | -.57164 |
|  | P_180+ | -.701130^*^ | .109966 | .000 | -1.04849 | -.35377 |
|  | AZunsep_180+ | -.081408 | .084319 | 1.000 | -.34354 | .18073 |
| AZunsep_180+ | M_180 | -.754425^*^ | .106909 | .000 | -1.09516 | -.41369 |
|  | P_180 | -.769145^*^ | .091424 | .000 | -1.05786 | -.48043 |
|  | AZ_180 | -.740905^*^ | .100513 | .000 | -1.06002 | -.42179 |
|  | M_180+ | -.958345^*^ | .146137 | .000 | -1.43294 | -.48375 |
|  | P_180+ | -.619722^*^ | .113282 | .000 | -.97697 | -.26248 |
|  | AZsep_180+ | .081408 | .084319 | 1.000 | -.18073 | .34354 |
| *. The mean difference is significant at the 0.05 level. | | | | | | |

**Table 22.** The statistical descriptives of middle lamella width of tissues before and after separation.

| Middle lamella width before and after separation | | | | | | | | |
| --- | --- | --- | --- | --- | --- | --- | --- | --- |
|  | N | Mean | Std. Deviation | Std. Error | 95% Confidence Interval for Mean | | Minimum | Maximum |
|  |  |  |  |  | Lower Bound | Upper Bound |  |  |
| M_180 | 20 | .92535 | .257135 | .057497 | .80501 | 1.04569 | .357 | 1.511 |
| P_180 | 20 | .99270 | .378224 | .084574 | .81569 | 1.16971 | .398 | 1.713 |
| AZ_180 | 25 | .94480 | .253818 | .050764 | .84003 | 1.04957 | .543 | 1.500 |
| M_180+ | 40 | .51940 | .165565 | .026178 | .46645 | .57235 | .237 | .900 |
| P_180+ | 20 | .58285 | .192137 | .042963 | .49293 | .67277 | .265 | .954 |
| AZsep_180+ | 30 | 1.48753 | .486282 | .088783 | 1.30595 | 1.66911 | .811 | 2.765 |
| AZunsep_180+ | 30 | 1.13390 | .268799 | .049076 | 1.03353 | 1.23427 | .645 | 1.720 |
| Total | 185 | .93544 | .442630 | .032543 | .87124 | .99965 | .237 | 2.765 |

**Table 23.** The test of homogeneity of variances of middle lamella width of tissues before and after separation. The Levene test rejects the hypothesis that the variances are equal.

| Middle lamella width before and after separation | | | |
| --- | --- | --- | --- |
| Levene Statistic | df1 | df2 | Sig. |
| 4.846 | 6 | 178 | .000 |

**Table 24.** The ANOVA test of middle lamella width of tissues before and after separation. The result rejects the hypothesis that there is no difference between the groups.

| Middle lamella width before and after separation | | | | | |
| --- | --- | --- | --- | --- | --- |
|  | Sum of Squares | df | Mean Square | F | Sig. |
| Between Groups | 19.806 | 6 | 3.301 | 36.171 | .000 |
| Within Groups | 16.244 | 178 | .091 |  |  |
| Total | 36.049 | 184 |  |  |  |

| **Table 25.** (Cont.) The Post Hoc tests of middle lamella width of tissues before and after separation by using DunnettT3. | | | | | | |
| --- | --- | --- | --- | --- | --- | --- |
| Middle lamella width before and after separation  Dunnett T3 | | | | | | |
| (I) Tissue_DAP | (J) Tissue_DAP | Mean Difference (I-J) | Std. Error | Sig. | 95% Confidence Interval | |
|  |  |  |  |  | Lower Bound | Upper Bound |
| M_180 | P_180 | -.067350 | .102267 | 1.000 | -.40053 | .26583 |
|  | AZ_180 | -.019450 | .076700 | 1.000 | -.26618 | .22728 |
|  | M_180+ | .405950^*^ | .063176 | .000 | .19664 | .61526 |
|  | P_180+ | .342500^*^ | .071776 | .001 | .10947 | .57553 |
|  | AZsep_180+ | -.562183^*^ | .105775 | .000 | -.90007 | -.22430 |
|  | AZunsep_180+ | -.208550 | .075593 | .155 | -.45121 | .03411 |
| P_180 | M_180 | .067350 | .102267 | 1.000 | -.26583 | .40053 |
|  | AZ_180 | .047900 | .098639 | 1.000 | -.27461 | .37041 |
|  | M_180+ | .473300^*^ | .088532 | .000 | .17490 | .77170 |
|  | P_180+ | .409850^*^ | .094860 | .004 | .09662 | .72308 |
|  | AZsep_180+ | -.494833^*^ | .122617 | .004 | -.88621 | -.10345 |
|  | AZunsep_180+ | -.141200 | .097781 | .950 | -.46112 | .17872 |
| AZ_180 | M_180 | .019450 | .076700 | 1.000 | -.22728 | .26618 |
|  | P_180 | -.047900 | .098639 | 1.000 | -.37041 | .27461 |
|  | M_180+ | .425400^*^ | .057116 | .000 | .24055 | .61025 |
|  | P_180+ | .361950^*^ | .066504 | .000 | .14869 | .57521 |
|  | AZsep_180+ | -.542733^*^ | .102271 | .000 | -.86974 | -.21573 |
|  | AZunsep_180+ | -.189100 | .070607 | .179 | -.41327 | .03507 |
| M_180+ | M_180 | -.405950^*^ | .063176 | .000 | -.61526 | -.19664 |
|  | P_180 | -.473300^*^ | .088532 | .000 | -.77170 | -.17490 |
|  | AZ_180 | -.425400^*^ | .057116 | .000 | -.61025 | -.24055 |
|  | P_180+ | -.063450 | .050310 | .986 | -.22735 | .10045 |
|  | AZsep_180+ | -.968133^*^ | .092562 | .000 | -1.26931 | -.66696 |
|  | AZunsep_180+ | -.614500^*^ | .055621 | .000 | -.79237 | -.43663 |
| P_180+ | M_180 | -.342500^*^ | .071776 | .001 | -.57553 | -.10947 |
|  | P_180 | -.409850^*^ | .094860 | .004 | -.72308 | -.09662 |
|  | AZ_180 | -.361950^*^ | .066504 | .000 | -.57521 | -.14869 |
|  | M_180+ | .063450 | .050310 | .986 | -.10045 | .22735 |
|  | AZsep_180+ | -.904683^*^ | .098632 | .000 | -1.22190 | -.58746 |
|  | AZunsep_180+ | -.551050^*^ | .065225 | .000 | -.75902 | -.34308 |
| AZsep_180+ | M_180 | .562183^*^ | .105775 | .000 | .22430 | .90007 |
|  | P_180 | .494833^*^ | .122617 | .004 | .10345 | .88621 |
|  | AZ_180 | .542733^*^ | .102271 | .000 | .21573 | .86974 |
|  | M_180+ | .968133^*^ | .092562 | .000 | .66696 | 1.26931 |
|  | P_180+ | .904683^*^ | .098632 | .000 | .58746 | 1.22190 |
|  | AZunsep_180+ | .353633^*^ | .101443 | .022 | .02927 | .67800 |
| AZunsep_180+ | M_180 | .208550 | .075593 | .155 | -.03411 | .45121 |
|  | P_180 | .141200 | .097781 | .950 | -.17872 | .46112 |
|  | AZ_180 | .189100 | .070607 | .179 | -.03507 | .41327 |
|  | M_180+ | .614500^*^ | .055621 | .000 | .43663 | .79237 |
|  | P_180+ | .551050^*^ | .065225 | .000 | .34308 | .75902 |
|  | AZsep_180+ | -.353633^*^ | .101443 | .022 | -.67800 | -.02927 |
| *. The mean difference is significant at the 0.05 level. | | | | | | |
