# Supplementary figures and images for: Cellular and Pectin Dynamics during Abscission Zone Development and Ripe Fruit Abscission of the Monocot Oil Palm
Source: Front Plant Sci. 2016 Apr 26;7:540. doi: 10.3389/fpls.2016.00540 (PMC4844998; doi:10.3389/fpls.2016.00540)

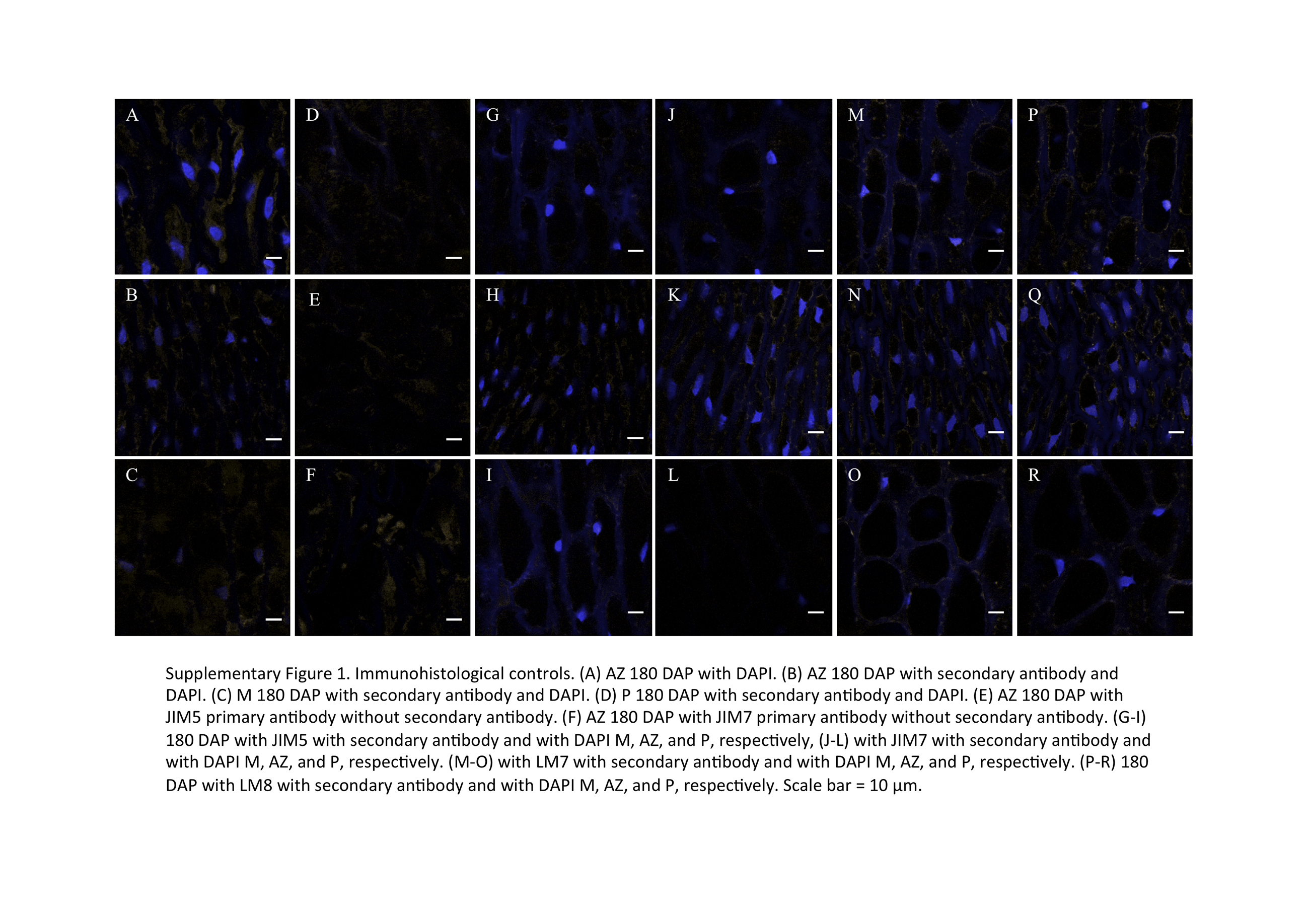

Supplement: Supplementary file 5 [file Image1.TIFF]

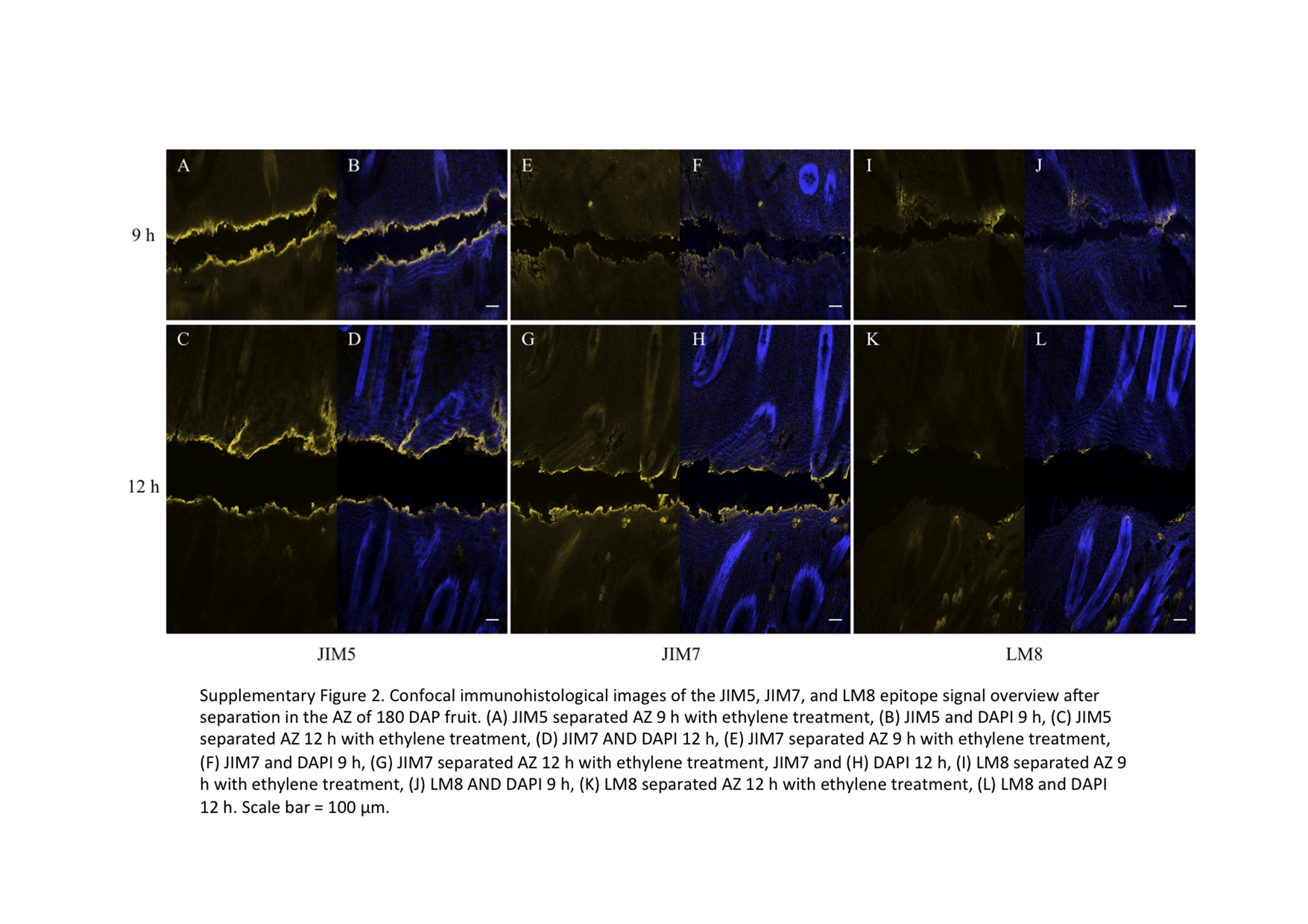

Supplement: Supplementary file 6 [file Image2.TIFF]

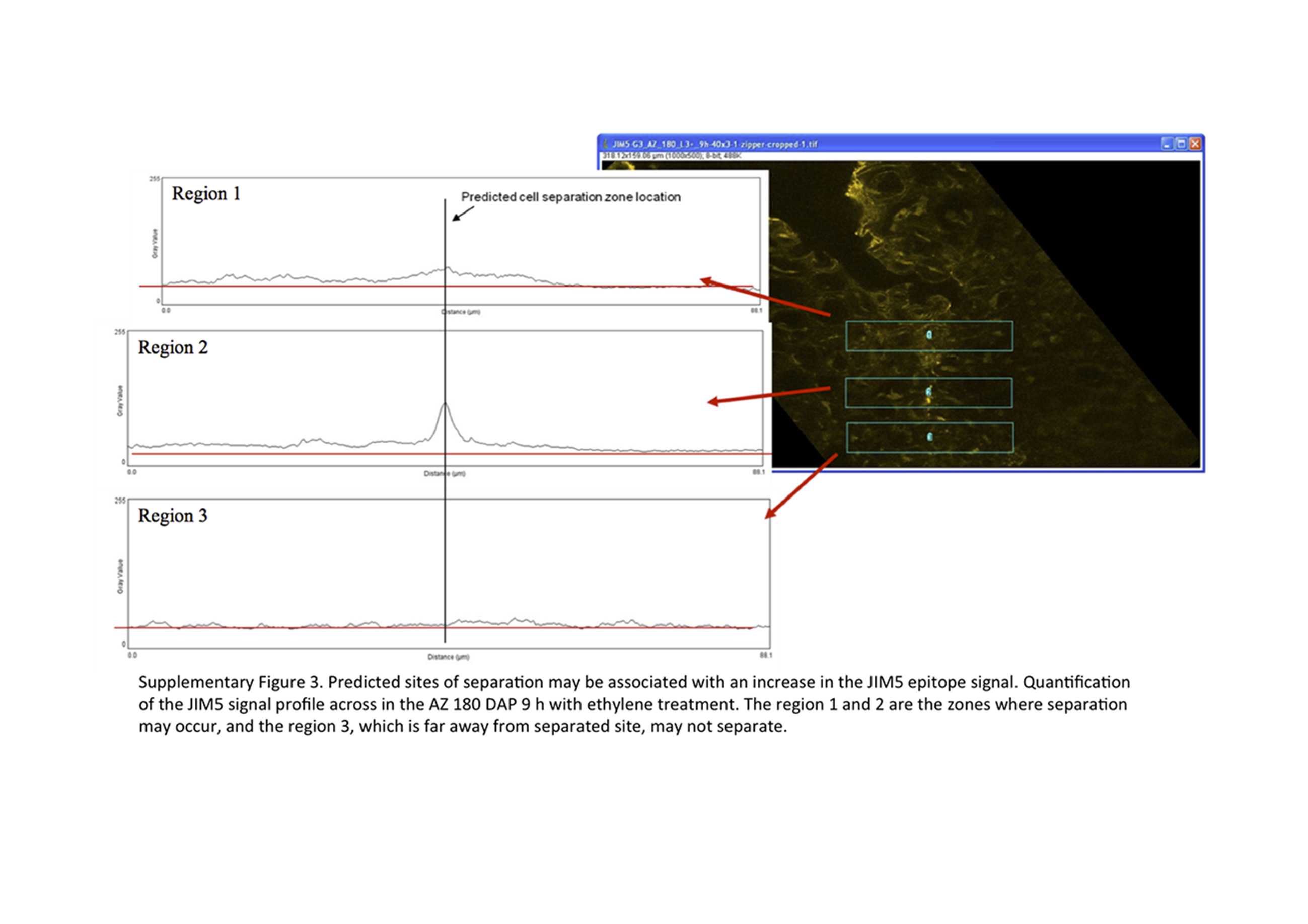

Supplement: Supplementary file 7 [file Image3.TIFF]

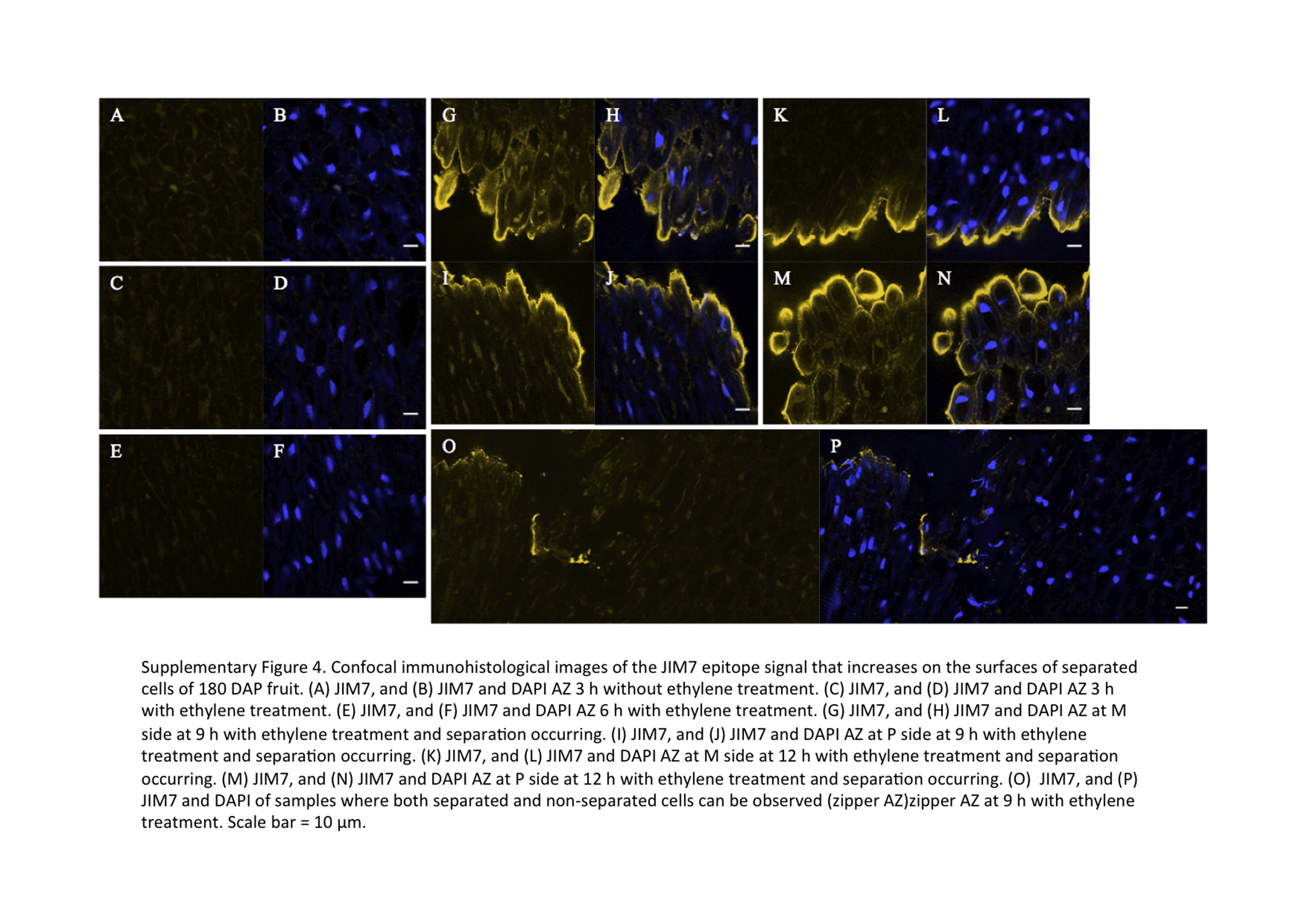

Supplement: Supplementary file 8 [file Image4.TIFF]

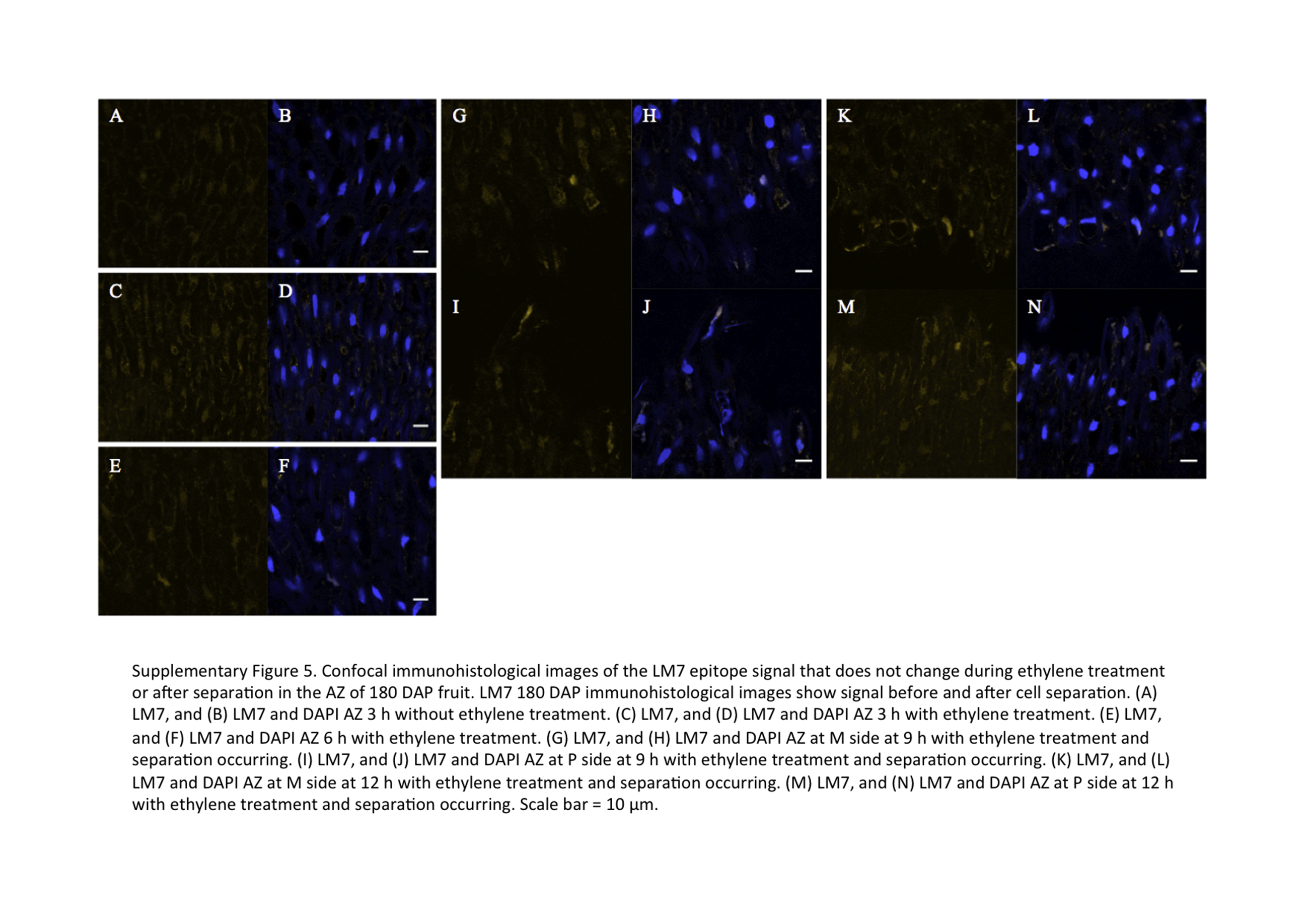

Supplement: Supplementary file 9 [file Image5.TIFF]

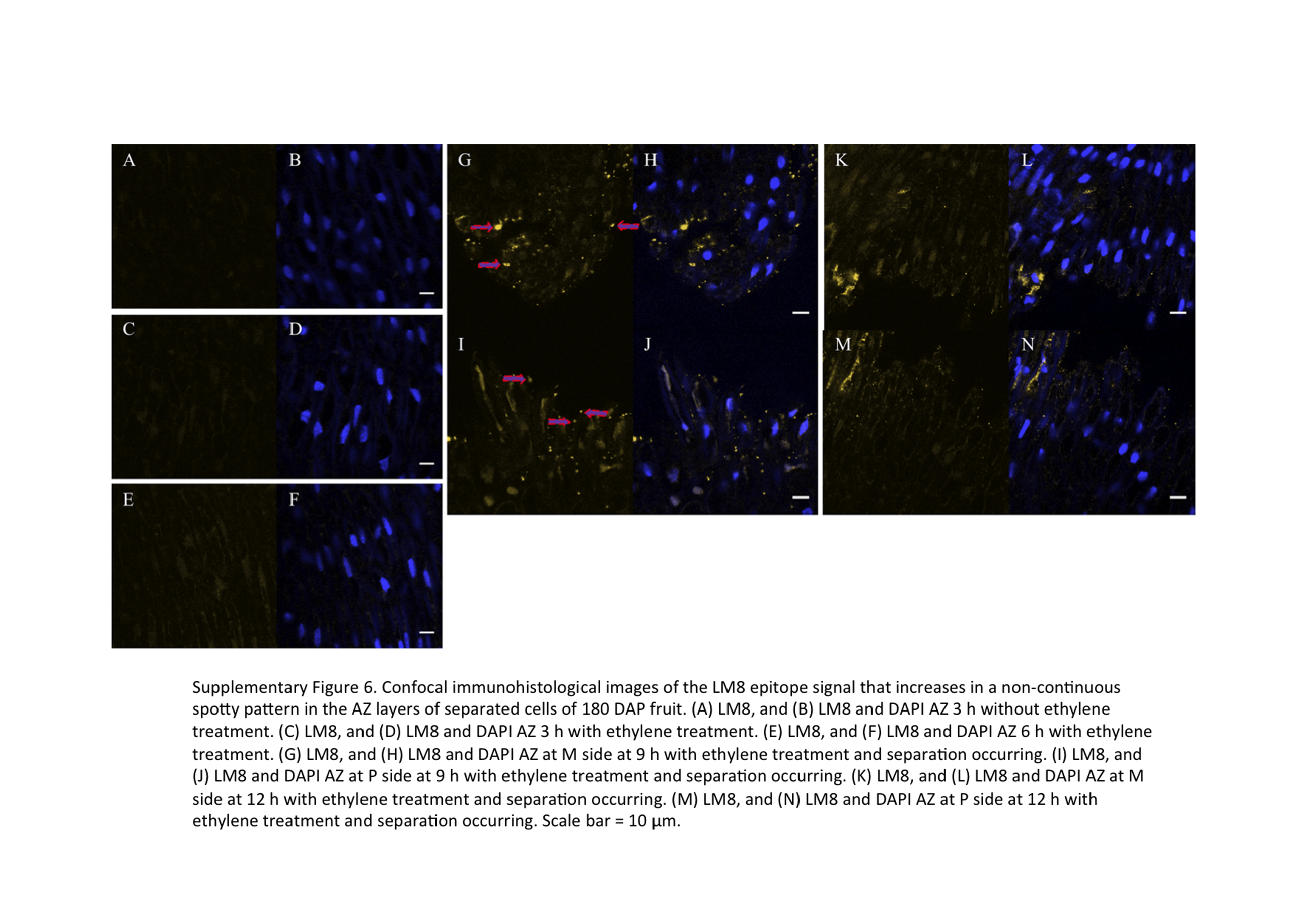

Supplement: Supplementary file 10 [file Image6.TIFF]
